# What is the effectiveness of printed educational materials on primary care physician knowledge, behaviour, and patient outcomes: a systematic review and meta-analyses

**DOI:** 10.1186/s13012-015-0347-5

**Published:** 2015-12-01

**Authors:** Agnes Grudniewicz, Ryan Kealy, Reitze N. Rodseth, Jemila Hamid, David Rudoler, Sharon E. Straus

**Affiliations:** Institute of Health Policy, Management and Evaluation, University of Toronto, Health Sciences Building, 155 College Street, Suite 425, Toronto, Canada; Li Ka Shing Knowledge Institute, St. Michael’s Hospital, 209 Victoria Street, 7th Floor, East Building, Toronto, Canada; Interactive Media Lab, Department of Mechanical and Industrial Engineering, University of Toronto, Bahen Centre for Information Technology, 40 St. George Street, Toronto, Canada; Perioperative Research Group, Department of Anaesthetics, Grey’s Hospital, Nelson R. Mandela School of Medicine, University of KwaZulu-Nata, Pietermaritzburg, South Africa; Department of Outcomes Research, Cleveland Clinic, Cleveland, OH USA; Clinical Epidemiology and Biostatistics, McMaster University, Hamilton, ON Canada

**Keywords:** Primary care, Evidence-based medicine, Printed educational materials

## Abstract

**Background:**

Printed educational materials (PEMs) are commonly used simple interventions that can be used alone or with other interventions to disseminate clinical evidence. They have been shown to have a small effect on health professional behaviour. However, we do not know whether they are effective in primary care. We investigated whether PEMs improve primary care physician (PCP) knowledge, behaviour, and patient outcomes.

**Methods:**

We conducted a systematic review of PEMs developed for PCPs. Electronic databases were searched for randomized controlled trials, quasi randomized controlled trials, controlled before and after studies, and interrupted time series. We combined studies using meta-analyses when possible. Statistical heterogeneity was examined, and meta-analysis was performed using a random effects model when significant statistical heterogeneity was present and a fixed effects model otherwise. The template for intervention description and replication (TIDieR) checklist was used to assess the quality of intervention description.

**Results:**

Our search identified 12,439 studies and 40 studies met our inclusion criteria. We combined outcomes from 26 studies in eight meta-analyses. No significant effect was found on clinically important patient outcomes, physician behaviour, or physician cognition when PEMs were compared to usual care. In the 14 studies that could not be included in the meta-analyses, 14 of 71 outcomes were significantly improved following receipt of PEMs compared to usual care. Most studies lacked details needed to replicate the intervention.

**Conclusions:**

PEMs were not effective at improving patient outcomes, knowledge, or behaviour of PCPs. Further trials should explore ways to optimize the intervention and provide detailed information on the design of the materials.

**Protocol registration:**

PROSPERO, CRD42013004356

**Electronic supplementary material:**

The online version of this article (doi:10.1186/s13012-015-0347-5) contains supplementary material, which is available to authorized users.

## Background

Printed educational materials (PEMs) are a simple, relatively inexpensive knowledge translation (KT) intervention for the dissemination of clinical information (such as clinical practice guidelines, journal articles, or evidence-based PDF or email summaries), aimed at improving the provision of care. A recent Cochrane review found that PEMs may have a small (0.02–0.13 standardized mean difference) beneficial effect on health professional practice outcomes [1]. However, despite continued publication of randomized controlled trials (RCTs) utilizing this intervention, we still know little about which behaviours can be influenced by PEMs, within which settings, and how to optimize the effect of these interventions for various health professionals.

Primary care physicians (PCPs) are required to have a vast and comprehensive knowledge base to treat different patient groups and diseases. On average, they have been observed to have 3.2 questions for every 10 patients they see [2] but these questions often go unanswered. PEMs are a potential strategy for meeting these needs. Non-interactive PEMs are easy to implement and scale across various primary care clinics. Reviews of the literature have found that printed resources (including books) remain a common source of information for physicians [3–5], with one systematic review finding that 50–80 % of physicians used printed materials for information [3]. However, if there is no demonstrated effectiveness of these interventions on knowledge, behaviour, or patient outcomes when targeted at PCPs, they should not be implemented as behaviour change techniques. To our knowledge, this is the first review to examine the effect of PEMs on PCPs.

Though interactive computer-based KT interventions such as those integrated within electronic health records have been shown to be effective in changing behaviour and are increasingly more popular than non-interactive paper-based interventions, they are expensive and require technological infrastructure and training, obstacles to implementation given limited budgets and overworked clinicians [6]. Surveys show that only 64 % of Canadian PCPs [7] and 41.5 % of American physicians [8] use electronic medical records, limiting the reach of complex interventions that are integrated into electronic records and possibly unintentionally leaving PCPs out of these interventions. With many different software vendors being used across practices (for example, there are 14 certified electronic medical record products to date in Canada alone [9]), creating a one-size-fits-all solution is challenging. As such, PEMs, a non-interactive and low-tech intervention, will likely continue to be used to disseminate new evidence and important clinical information or as a part of multi-component KT interventions.

The objective of this review was to examine what effect PEMs have on PCP knowledge, behaviour, and patient outcomes, in comparison to no intervention or to other single- or multi-component educational interventions. This review contributes to existing literature by examining the effect of interventions specifically designed for PCPs. The primary care setting is considerably different from other health care settings, and PCPs are likely to experience barriers unique to their setting and their scope of practice. Physicians are the population of interest to limit participant heterogeneity as we anticipated that differences in training and role among diverse primary care clinicians may influence behaviour change. PEMs for PCPs may have different content and may target different behaviour than PEMs for other professionals. More importantly, PCPs may respond differently than other clinicians to PEMs, and we anticipate PEMs have a different effect size when targeting behaviour change in different providers. We also examined the quality of reporting of PEM interventions in included studies.

## Methods

A systematic review protocol was written for this review and registered with PROSPERO, the international prospective register of systematic reviews (registration no. CRD42013004356). We based the methods for this review on those described in the Cochrane Handbook for Systematic Reviews of Interventions and the Cochrane Effective Practice and Organisation of Care Group (EPOC) [10]. It is reported using the PRISMA Statement for Reporting Systematic Reviews [11].

### Eligibility criteria

We included studies reporting the effectiveness of PEMs for PCPs (family physicians as well as specialists practising primary care such as pediatricians), defined for the purpose of the review as guidelines, summaries of guidelines, the dissemination of published or non-published information, recommendations, or evidence presented in print or electronic form. Electronic materials include PDFs, other document files (e.g. Microsoft Word documents), and non-interactive web pages. Studies examining interactive online educational materials (such as online courses) or patient-specific materials were excluded. We included any method of delivery of the intervention (e.g. email, mail, fax) or level of intensity (i.e. how often the intervention was delivered). We limited study designs to RCTs, quasi randomized trials, controlled before and after studies, and interrupted time series (ITS) analyses. We included reported patient outcomes, physician cognition (skills and knowledge), and physician behaviour outcomes. Physician attitudes were not included in the review. No restrictions were placed on publication status or date of publication. Studies not published in English were excluded. Lastly, comparisons had to allow for the isolation of the effect of PEMs on outcomes, meaning that studies comparing two PEMs with no control group or studies comparing two interventions where both groups included PEMs were excluded. Researchers independently and in duplicate screened each title and abstract (AG, SR, RK, DR) and reviewed the full text of selected studies for eligibility using the criteria listed above (AG, SR, RK).

### Information sources and search

Studies were identified by a search of electronic databases developed by an information specialist and independently reviewed by a second information specialist. Medline (Ovid MEDLINE(R)), EMBASE (Embase Classic + Embase), ERIC (ProQuest), and the Cochrane Central Register of Controlled Trials were searched on November 25, 2014. The following search terms were used: print, message, book, monograph, pamphlet, journal, educational materials, online, email, web-based, general practitioner, and family physician (see Additional file 1 for Medline search). Appropriate wildcards were used in the search to account for plurals and variations in spelling. The search was supplemented by searching the reference lists of included articles and of other systematic reviews. Authors of studies published within the last 10 years were contacted to collect missing data needed for meta-analyses.

### Data extraction

We extracted data independently and in duplicate (AG, RK) using a modified version of the Cochrane EPOC standardized data collection checklist [10]. A calibration exercise was done before screening and data extraction with each of the researchers to ensure consistency. Information was extracted from each study on study design, the intervention, controls, type of targeted behaviour, professional and patient participants, setting, methods, outcomes, costs of the intervention, changes in healthcare costs, and results. All disagreements for screening and extraction were resolved by discussion.

### Risk of bias

Risk of bias was assessed independently and in duplicate using the Cochrane EPOC risk of bias assessment tool [10].

### Data synthesis

Clinical (e.g. type of population, topic of intervention), methodological (e.g. study duration), and statistical heterogeneity were assessed. For studies with similar clinical and methodological characteristics that reported similar outcomes, data were pooled statistically using meta-analysis, with separate meta-analyses carried out for patient outcomes and for physician outcomes. Physician outcomes were grouped post hoc according to Miller’s framework for clinical assessment into physician behaviour (“does” and “shows how”) and physician cognition (“knows” and “knows how”). Miller’s framework presents four categories, with knowledge (“knows”) at the base to represent that a physician knows what is required to effectively carry out professional functions. Following knowledge is competence, or “knows how”, which captures the skills needed to acquire information, analyze and interpret data, and translate findings into their practice. Performance (“shows how”) follows, where physicians must demonstrate their knowledge within an examination setting, and finally is action (“does”) which is the independent action of the physician in clinical practice [12]. We then grouped the studies within these categories into either binary or continuous outcomes. Only one outcome per study was used in any single meta-analysis to avoid double counting. The study’s primary outcome was chosen when possible. When more than one primary or eligible outcome existed, the outcome included in the meta-analysis was chosen at random. A meta-analysis was conducted when outcomes from two studies could be pooled using R software and the Metafor Package [13, 14].

Statistical heterogeneity was examined using the *I*^2^ statistic. A random-effects meta-analysis was conducted when heterogeneity was statistically significant; otherwise, a fixed-effects model was used. When *I*^2^ was significant, we conducted post hoc sub-analyses to explore the source of heterogeneity. For continuous outcomes, a standard mean difference (SMD) was calculated because outcomes were measured using different scales. For binary outcomes, a relative risk (RR) was used as an effect measure. Studies were adjusted for clustering when applicable using study-reported intracluster coefficients (ICCs). When not provided in the study, an ICC was selected from the University of Aberdeen Database of ICCs [15]. When a range of possible ICCs was provided in the literature, sensitivity analysis was performed using multiple adjustments to determine sensitivity of the pooled estimates with respect to ICC values. For meta-analyses of continuous outcomes, when a standard deviation (SD) or standard error was not provided, it was imputed from similar outcomes within the study. Sensitivity analysis was also performed with respect to the imputed standard errors. Studies that did not provide sufficient data for the calculation of a summary statistic were not included in the meta-analysis. A narrative description was used to present the data that could not be pooled.

### Intervention reporting

Studies were assessed against the template for intervention description and replication (TIDieR checklist) to determine the completeness of reporting and replicability of interventions in the included studies [16]. The checklist was applied from the perspective of the intervention of interest for our review, which may have been one of several interventions tested or used as control.

## Results

### Literature search

**Fig. 1:**
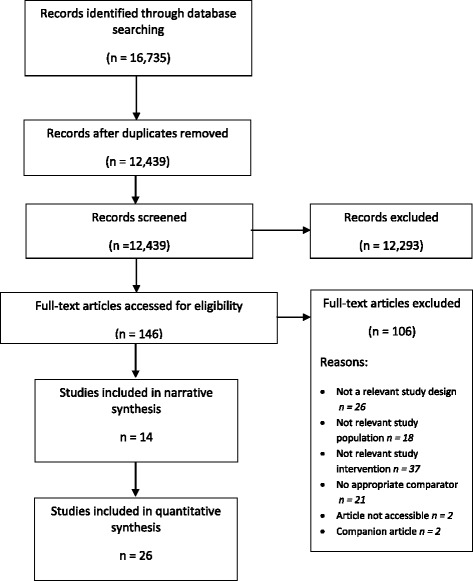
Identification of eligible studies.

From the 16,735 articles retrieved, the literature search resulted in 12,439 citations (duplicates removed) and 146 potentially relevant citations. Forty studies and two companion reports met eligibility criteria. See Fig. 1 for a flow diagram representing identification of eligible studies [11].

### Study characteristics

Thirty-seven of the included studies were RCTs, and three were studies with an ITS design. Studies were conducted between 1983 and 2014 in the USA [17–27], UK [28–36], Canada [37–44], Australia [45–47], Germany [48–50], the Netherlands [51], Denmark [52], Brazil [53], Switzerland [54], Norway [55], and Italy [56]. A wide range of intervention topics was observed in our systematic review. Study characteristics are outlined in Table 1.

**Table 1:** Characteristics of included studies

Author (year)	Study type	Country	Study topic	Intervention	Comparison	# of physicians	Behaviours targeted
Avorn, J. (1983)	RCT	USA	Drug prescribing	Recommendations	Another intervention, usual care	435	Physician behaviour
Bearcroft, P.W.P. (1994)	RCT	England	Medical imaging	Full guideline or summary	Usual care	210	Physician behaviour
Bishop, P. (2006)	RCT	Canada	Low back pain	Whole guideline	Another intervention, usual care	462	Physician behaviour
Bjornson, D.C. (1990)	RCT	USA	Hypertension/cardiovascular illness	Journal article	Usual care	576	Physician behaviour
Butzlaff, M. (2003)	RCT	Germany	General	Whole guideline	Usual care	72	Physician cognition
Denig, P. (1990)	RCT	Netherlands	Irritable bowel syndrome and renal colic	Bulletin	Usual care	209	Physician behaviour, physician cognition
Dickinson, W.P. (2003)	RCT	USA	Mental illness	Recommendations	Usual care	Not clear	Patient
Dormuth, C.R. (2004)	RCT	Canada	Drug prescribing	Bulletin	Usual care	499	Physician behaviour
Downs, M. (2006)	RCT	UK	Dementia	Electronic case analysis	Another intervention, usual care	Not clear	Physician behaviour
Dubey, V. (2006)	RCT	Canada	Prevention	Checklist with recommendations	Usual care	38	Physician behaviour
Evans, C.E. (1986)	RCT	Canada	Hypertension/cardiovascular illness	Educational/Informational Package	Usual care	76	Patient, physician behaviour
Feng, B. (2013)	RCT	USA	Prostate cancer screening	Recommendations	Another intervention	118	Physician behaviour
French, S. (2013)	RCT	Australia	Low back pain	Whole guideline	Another intervention	92	Physician behaviour, physician cognition
Guadagnoli, E. (2004)	RCT	USA	Hypertension/cardiovascular illness	Recommendations	Usual care	247	Physician behaviour
Guthrie, B. (2013)	ITS	Scotland	Dementia	Recommendations	N/A	Not clear	Physician behaviour
Hazard, R.G. (1997)	RCT	USA	Low back pain	Algorithm	Usual care	30	Patient
Hunskaar, S. (1996)	RCT	Norway	Drug prescribing	Recommendations	Usual care	374	Physician cognition
Kottke, T.E. (1989)	RCT	USA	Smoking cessation	Manual	Another intervention, usual care	66	Patient, physician behaviour
Kunz, R. (2007)	RCT	Germany	Drug prescribing	Recommendations	Usual care	132	Physician behaviour
Liaw, S.T. (2008)	RCT	Australia	Asthma	Guideline summary	Another intervention, usual care	51 total	Physician behaviour, physician cognition
Matowe, L. (2002)	ITS	Scotland	Medical imaging	Whole guideline	N/A	376	Physician behaviour
McEwan, A. (2002)	RCT	England	Smoking cessation	Educational/informational package	Usual care	107	Physician behaviour
Mukohara, K. (2005)	RCT	USA	General	Journal summary	Another intervention	107	Physician cognition
Nicholas, J. (2009)	RCT	USA	Obesity	Recommendations	Usual care	1000	Physician behaviour
Oakeshott, P. (1994)	RCT	England	Medical imaging	Sections of a guideline	Usual care	170	Physician behaviour
Perria, C. (2007)	RCT	Italy	Diabetes	Whole guideline	Another intervention, usual care	252	Physician behaviour
Rabin, D. (1994)	RCT	USA	Sexually transmitted diseases	Educational/informational package	Another intervention, usual care	961	Physician behaviour
Rahme, E. (2005)	RCT	Canada	Osteoarthritis	Recommendations	Another intervention, usual care	249	Physician behaviour
Secher, N. (2012)	RCT	Denmark	Life support	Poster	Usual care	830	Physician cognition
Shah, B. (2014)	RCT	Canada	Diabetes and cardiovascular disease	Educational/informational package	Usual care	Not clear	Patient, physician behaviour
Simon, A.E. (2010)	RCT	Switzerland	Mental illness	Clinical vignette	Usual care	1138	Physician cognition
Szonyi, G. (1994)	RCT	Australia	Incontinence	Educational/informational package	Usual care	124	Physician cognition
Tsuji, S.R. (2007)	RCT	Brazil	Mental illness	Educational/informational package	Usual care	8	Patient, physician behaviour
Tziraki, C. (2000)	RCT	USA	Prevention	Manual	Another intervention, usual care	810	Physician behaviour
Ulbricht, S. (2014)	RCT	Germany	Psychotropic drug use	Manual	Usual care	852	Physician behaviour
Watson, E. (2001)	RCT	England	Genetic services for breast cancer	Educational/informational package	Another intervention, usual care	688	Physician cognition
Watson, M. (2001)	RCT	England	Drug prescribing	Recommendations	Another intervention, usual care	107	Physician behaviour
Worrall, G. (1999)	RCT	Canada	Mental illness	Whole guideline	Another intervention	42	Patient, physician behaviour
Wright, N.M.J. (2004)	ITS	England	Drug prescribing	Recommendations	N/A	444	Physician behaviour
Zwarenstein, M. (2014)	RCT	Canada	Diabetes and retinal screening	Recommendations	Usual care, variations in intervention	5048	Physician behaviour

Very few studies provided details on the study population such as age and time since graduation. Fourteen studies provided information on age of the participants, and nine studies provided information on time since graduation from medical school. The number of physician participants in each study ranged from 8 to 5048. All studies were composed of a majority of family physicians, with some studies including pediatricians [24], general internists [17, 20, 26, 40, 48], and a small number of other specialists [17, 20, 48]. Three [19, 28, 47] of the studies included in this review examined the cost of the intervention, with two studies [31, 47] calculating the direct impact of the interventions on health care costs. Avorn and Soumerai [19] found a cost reduction of $105 (US dollars) per physician over 9 months for their academic detailing intervention but did not calculate any savings for the PEM (used as control). Watson et al. [28] calculated the costs of the guideline intervention and found no significant reduction in prescribing costs. French et al. [47] conducted a cost-effectiveness analysis that found that a more active intervention (including a workshop) was less expensive than the existing standard PEM dissemination strategy. However, savings in health gains and service reductions were not sufficient to make the active strategy cost-effective. No studies looked at changes in non-health care costs.

### Comparisons

Of the RCTs, 21 studies compared PEMs to usual care [17, 18, 21, 22, 24, 29, 30, 35, 38, 40–42, 45, 48–55], 12 to both usual care and another intervention [19, 20, 23, 25, 28, 33, 34, 39, 43, 44, 46, 56], and four to another intervention [26, 27, 37, 47].

### Intervention details

Several types of interventions were included such as guidelines [32, 37, 44, 47, 49, 56], guideline summaries or sections of guidelines [29, 46], practice recommendations [19, 21, 22, 24, 27, 28, 31, 36, 39, 43, 48, 55], educational or information packages [20, 34, 35, 40, 42, 45, 53], manuals [23, 25, 50], bulletins [38, 51], clinical vignettes [57], a journal summary [26], a checklist with recommendations [41], an electronic case analysis [33], an algorithm [18], a poster [52], and a journal article [17]. One study was not clear whether the intervention was a complete guideline or summary [30].

### Reporting of interventions (TIDieR checklist)

All studies defined or provided a name for their intervention and the majority (80 %, *N* = 32) of the studies provided a rationale, theory, or goal of the intervention. All but three studies described the materials in the intervention; 32.5 % (*N* = 13) of the studies made the materials available in the paper or in supplementary materials or websites. Two studies provided web links or references to the PEMs that did not work or were not available; these were counted as materials not provided [18, 26]. Thirty-three studies described the intervention procedures, 35 described the location of the intervention, and 26 described the timeframe and number of times the intervention was delivered. Fifteen percent (*N* = 6) of the studies addressed planned measurement of adherence and 22.5 % (*N* = 9) studies measured adherence to the intervention (e.g. receipt of materials, reading of materials). Details on reporting of interventions are provided in Additional file 2.

### Outcomes measured and behaviour targeted

Eight studies measured one or more patient outcomes as a result of the PCP-targeted intervention [18, 21, 25, 37, 40, 42, 46, 53]. The majority of included studies (77.5 %, *N* = 31) measured physician behaviour outcomes [17, 19, 20, 22–25, 27–33, 35–42, 44, 46–48, 50, 51, 53, 56, 58], and 25 % (*N* = 10) of the studies measured physician cognition outcomes [26, 34, 45–47, 49, 51, 52, 54, 55]. Additional File 3.

### Risk of bias of included studies

**Fig. 2:**
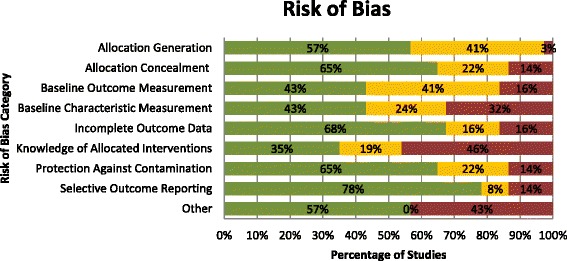
Risk of bias— Legend: *green*: low, *yellow*: unclear, *red*: high

Using the Cochrane EPOC risk of bias assessment tool, 33 RCTs had unclear or high risk of bias for at least two criteria. Two RCTs were appraised as low risk of bias on eight of nine criteria [38, 43], and only two RCTs were appraised as having low risk of bias on all nine criteria [41, 42] (See Additional file 4). The ITS studies had at least two unclear risk of bias out of seven criteria. Figure 2 illustrates the percentage of studies at low, high, and uncertain risk of bias.

### Meta-analyses

Eight meta-analyses were conducted. Results are presented in Table 2, and forest plots are available in Additional File 5.

**Table 2:** Results of meta-analyses

Outcome	Number of studies	Summary statistic	Confidence interval	*I* ^2^ statistic (%)
		RR = relative risk		
		SMD = standard mean difference		
Patient outcomes				
Dichotomous patient outcomes	5	RR = 1.00	1.00–1.00	0
Dichotomous patient outcomes (sensitivity analysis with Shah 2014 removed)	4	RR = 1.09	0.91–1.29	0
Physician behaviour outcomes				
Continuous physician behaviour outcomes (*N* = physicians)	4	SMD = 0.35	−0.06–0.76	67.88
Continuous physician behaviour outcomes (*N* = patients)	3	SMD = 0.27	−0.03–0.57	68.74
Dichotomous physician behaviour outcomes (*N* = physicians)	3	RR = 1.01	0.96–1.07	0
Dichotomous physician behaviour outcomes (*N* = patients)	9	RR = 0.99	0.97–1.01	0
Dichotomous physician behaviour outcomes (PEM vs. workshop)	2	RR = 0.57	0.12–2.73	64.48
Physician cognition outcomes				
Continuous physician cognition outcomes	3	SMD = 0.65	−0.21–1.51	89.75
Dichotomous cognition outcomes	3	RR = 1.51	0.90–2.52	90.27

#### Patient outcomes

A meta-analysis of dichotomous patient outcomes was conducted with five studies and 935,252 patients [18, 25, 40, 42, 53], combined using a fixed-effects model. The summary statistic demonstrated no improvement with use of a PEM, RR = 1.00 (95 % CI = 1.00,1.00, *I*^2^ = 0). One study [42] was allocated 100 % of the weight in the analysis; therefore, we completed a sensitivity analysis without the study for a total of 1463 patients, which resulted in a small but statistically insignificant improvement, RR = 1.09 (95 % CI = 0.91,1.29, *I*^2^ = 0).

#### Physician behaviour outcomes

Five meta-analyses of studies reporting impact on physician behaviour outcomes were conducted. A meta-analysis of continuous physician outcomes was conducted with four studies and 531 physicians [24, 25, 28, 35]. The results were not statistically significant, SMD = 0.35 (95 % CI = −0.06, 0.76, *I*^2^ = 67.88 %). Sub-analyses grouping studies by similar topic, type of PEM, length of intervention, type of participants, and the behaviour targeted found that the PEM topic was the largest source of heterogeneity (see Additional File 6). Three studies assessing impact on continuous physician outcomes per patient including 1106 patients were combined [22, 33, 41]. No statistically significant result was found, SMD = 0.27 (95 % CI = −0.03, 0.57, *I*^2^ = 68.74 %). Sub-analyses grouping studies by similar type of intervention, risk of bias, and behaviour targeted found that the behaviour targeted was the largest source of heterogeneity.

A meta-analysis of dichotomous physician behaviour outcomes was conducted with results from three studies including 999 physicians [20, 46, 50]. No statistically significant improvement was observed with the use of PEM, RR = 1.01 (95 % CI = 0.96, 1.07, *I*^2^ = 0.00 %). Nine studies assessing impact of PEMs on dichotomous physician behaviour outcomes per patient including 3,273,788 patients were combined [22, 38, 40, 42, 44, 48, 53, 56, 58]. No statistically significant improvement was observed with PEM, RR = 0.99 (95 % CI = 0.97, 1.01, *I*^2^ = 0.00 %). Two studies assessing dichotomous physician behaviour outcomes of PEMs compared to workshops were combined including 153,089 patients [37, 47]. No statistically significant difference was found between the two interventions, RR = 0.57 (95 % CI = 0.12, 2.73, *I*^2^ = 64.48 %).

#### Physician cognition outcomes

Three studies (438 physicians) were combined in a meta-analysis of studies reporting continuous physician cognition outcomes [45–47]. No statistically significant effect was found, SMD = 0.65 (95 % CI = −0.21, 1.51, *I*^2^ = 89.75 %). Three studies (806 physicians) were combined in a meta-analysis of dichotomous physician cognition outcomes [34, 51, 52], and no significant effect was found, RR = 1.51 (95 % CI = 0.90, 2.52, *I*^2^ = 90.27 %). The high statistical heterogeneity for both cognitive meta-analyses could not be examined. Subgroup analyses could not be performed as all studies varied in topic, method of knowledge measurement, type of intervention, and length of intervention.

### Narrative synthesis

Fourteen studies examined 71 outcomes that were not included in the meta-analyses due to heterogeneity in study outcomes (i.e. outcomes reported could not be pooled with any other study), study design (i.e. ITS studies were not pooled with RCTs), or missing data (i.e. studies did not provide sufficient data and authors could not be reached) (see Additional file 3 for all study outcomes). The results are presented below by type of outcome.

#### Patient outcomes

One study [21] examined the effect of PEMs on four clinical patient outcomes compared to usual care. A significant effect was found for only one outcome (physical functioning of patients with multisomatoform disorder).

#### Physician behaviour

Nine studies [17, 19, 23, 29–32, 36, 39] examined the effect of PEMs on 55 physician behaviour outcomes compared to usual care. Bearcroft et al. [30] found a significant effect of PEMs on all four outcomes studied in chest radiography referral, including concordance with guidelines and documentation of history, clinical diagnosis, and smoking history. Guthrie et al. [36] found a significant effect of PEMs on six out of 12 outcomes examining prescriptions of antipsychotics. Oakeshott et al. [29] found a significant improvement with PEMs on total radiology requests but not when examined by X-ray type (limbs, chest, or spine). Worrall et al. [37] found a significant improvement with PEMs on two of five prescribing outcomes. No effect was found on outcomes in five other studies [17, 19, 23, 32, 39].

One study [27] examined the effect of PEMs compared to a web course on eight physician behaviour outcomes. There was a significant difference between the two interventions for three of the eight outcomes (overall shared decision-making, guiding in decision-making, stating that a PSA test would be ordered).

#### Physician cognition

Three studies [26, 49, 55] examined four physician cognition outcomes. None of the studies found an improvement in cognition after a PEM intervention.

## Discussion

We included 40 studies and conducted eight meta-analyses with data from 26 studies. The reported quality of evidence ranged from low to high and many studies were unclear on important methodological factors such as allocation sequence generation (15 studies unclear), baseline outcome measurement (15 studies unclear), and baseline characteristic measurement (nine studies unclear). No significant effect was seen across the eight meta-analyses. However, six of the meta-analyses had three or fewer studies included.

Statistical heterogeneity was high for both physician cognition meta-analyses, possibly due to different ways of measuring knowledge outcomes, the different topic areas, duration of the intervention, risk of bias, or the type of PEM intervention. Clinical heterogeneity was visible across studies in all meta-analyses as the topic of the interventions varied greatly. Behaviour change of PCPs may vary by clinical area addressed by the intervention. Though heterogeneity in the population was limited by the review’s focus on PCPs, it was difficult to determine how the population varied across studies due to limited reporting on participant age, time since graduation, and academic affiliation. Methodological heterogeneity was also a concern as study durations varied widely and many factors could not be assessed such as how the intervention was delivered, the source of the evidence, setting of the participant’s clinical practice, and participant demographic details.

The narrative synthesis examined 71 study-level outcomes across three outcome categories. When compared to usual care, PEMs resulted in significant improvement in outcomes for one of four clinical patient outcomes, 13 physician behaviour outcomes, and no physician cognition outcomes. Though it is difficult to draw conclusions from the narrative syntheses of these outcomes, it is evident that a large majority of the measured outcomes did not statistically improve as a result of PEMs. These results differ from the review by Giguère and colleagues [1], which included all health professionals, and found a median improvement in SMD for categorical practice outcomes of 0.02 and a median improvement in SMD for continuous professional outcomes of 0.13 when compared to usual care. Only 11 of our 40 studies overlapped with the Giguère review. Our results suggest that the effect found in the review by Giguère and colleagues may not be applicable to PCPs and that the effect was a result of interventions targeted at specialist physicians. Most of the studies included in the Cochrane review (42 of 45) included physicians, though the clinical speciality was not clear in many of the included studies and no subgroup analyses were conducted by type of health professional. The difference in effect size may be a result of differences in PEM content between PCP and other clinician PEMs, in the evidence needs of PCPs, in the practice context, or in the behaviours targeted by the PEM. The methods used to synthesize data in the Cochrane review included the use of more than one outcome per study and may have resulted in an inflated effect size. The authors of the Cochrane review also used a different approach when more than one measure was provided in a study by abstracting the median measure whereas we used a random selection process. Furthermore, our review builds on the existing literature as it examines the intervention’s effect on knowledge, an outcome that was excluded from the Cochrane review.

Though it is assumed that the cost of these interventions is small relative to interactive educational tools or active evidence-dissemination strategies, the costs need to be systematically assessed. Three of the studies included in this review provided an economic analysis, but two of the three studies provided costs for a more active intervention and not for the control PEM. Before widely disseminating any behaviour change intervention, information on cost effectiveness of the intervention is critical.

### Intervention reporting TIDieR checklist

One study [48] met all criteria of the TIDieR checklist, and three studies met all but one of the 12 criteria [41, 43, 49]. This is a concerning finding given the relative simplicity of PEM interventions. However, it may be this perceived simplicity that has discouraged study authors from describing the intervention and its development. Improvement in intervention reporting is needed, such as providing access to the intervention materials through appendices or permanent web links. Most study authors noted that they did not know if PEMs were received and read, a significant limitation of the included studies. Other key areas for improvement in reporting of interventions are detailing who provided the intervention, describing how the intervention was delivered, and specifying the date the intervention was delivered.

### Limitations

Differences in outcome measurement and limited reporting resulted in the inability to pool the results for a number of studies. A small number of trials were eligible for each meta-analysis, with one of the eight meta-analyses only pooling two studies, limiting our ability to conduct a meta-regression to explore effect modifiers. It is possible that the meta-analyses had too few included outcomes to detect the small differences that may be expected from PEMs. The physician cognition meta-analyses may be limited in their usefulness due to high statistical heterogeneity. The review may also have been limited by only including English-language studies.

## Conclusions

This systematic review has found that PEMs do not improve patient, PCP behaviour, or PCP knowledge outcomes. A previous Cochrane systematic review of 45 studies (14 RCTs and 31 ITSs) noted that similar, passive dissemination strategies have small to moderate effects (1). However, the Cochrane review included all health care professionals, excluded knowledge outcomes, and combined more than one outcome per study (possibly inflating the result) in its meta-analyses.

These findings are of particular importance to those interested in knowledge translation (guideline developers, quality agencies, government agencies, pharmaceutical companies, etc.) and in developing, disseminating, and evaluating PEM interventions. Individuals and groups interested in developing PEMs should note that in their current state, there is no evidence that PEMs are effective at improving PCP outcomes. However, it is difficult to ascertain whether the PEMs tested have been optimized and whether adequate reach of the intervention has been achieved. There are too few details in published studies on how these materials were developed (use of theory, use of evidence-based design, involvement of the end-user) and too limited descriptions of the materials. The results also have implications for research funding bodies. Continuing to fund studies on PEMs with little description of the intervention, inadequate power, or a lack of optimization of the intervention is a poor use of resources. Researchers interested in conducting future studies of PEMs for primary care should invest resources in better design of the tools and should provide detailed descriptions of the intervention to determine if PEM optimization can result in improved outcomes. Trials should be sufficiently powered to detect the small effect of these interventions. Head to head studies of different designs of PEMs may help us understand how certain design elements may contribute to effectiveness, though these studies should continue to include usual care comparisons. Otherwise, we should accept the role of PEMs as simple tools for the dissemination of information or as a part of other more interactive interventions, rather than as tools to influence knowledge and behaviour of PCPs.

## Additional file

Additional file 1:
**Medline Search.** Search strategy for the Medline database. (PDF 142 kb) Additional file 2:
**TIDieR Checklist.** Details on reporting of interventions. (PDF 204 kb) Additional file 3:
**Study Outcomes.** All study outcomes for included studies. (PDF 543 kb) Additional file 4:
**Risk of Bias.** Appraisal of risk of bias of included RCT studies using Cochrane EPOC risk of bias assessment tool. (PDF 141 kb) Additional file 5:
**Meta-Analysis Results.** Forest Plots for Meta-Analyses. (PDF 395 kb) Additional file 6:
**Sub-analyses for Heterogeneity.** Results of sub-analyses for heterogeneity. (PDF 140 kb)

## Abbreviations

PCP: primary care physician
PEMs: printed educational materials
RCT: Randomized controlled trials
ITS: Interrupted time series
SMD: Standard mean difference
RR: Relative risk
ICC: Intracluster correlation coefficient
SD: Standard deviation
