# Supplementary material for: What is the effectiveness of printed educational materials on primary care physician knowledge, behaviour, and patient outcomes: a systematic review and meta-analyses
Source: Implement Sci. 2015 Dec 1;10:164. doi: 10.1186/s13012-015-0347-5 (PMC4666153; doi:10.1186/s13012-015-0347-5)
Supplement: Additional file 1: — Medline Search. Search strategy for the Medline database. (PDF 142 kb) [file 13012_2015_347_MOESM1_ESM.pdf]

Additional file 1: Medline Search

Database: Ovid MEDLINE(R), Ovid MEDLINE(R) In-Process & Other Non-Indexed Citations, Ovid MEDLINE(R) Daily and Ovid OLDMEDLINE(R) <1946 to Present>
Search Strategy:
----

1 Physicians, Family/ [Primary Care] (14041)
2 Family Practice/ (57860)
3 (general adj practice$).mp. (33082)
4 (family adj practice$).mp. (60308)
5 (family adj medicine).mp. (5825)
6 (community adj practice$).mp. (1071)
7 (group adj practice$).mp. (10040)
8 (institution$ adj practice$).mp. (1575)
9 (physician$ adj practice$).mp. (35997)
10 (private adj practice$).mp. (11602)
11 (solo adj practice$).mp. (404)
12 General Practitioners/ (677)
13 Hospitalists/ (1125)
14 Occupational Health Physicians/ (35)
15 Osteopathic Physicians/ (93)
16 Physicians, Primary Care/ (427)
17 (community adj (doctor? or physician? or practitioner?)).mp. (897)
18 (family adj (doctor? or physician? or practitioner?)).mp. (14713)
19 (general adj (doctor? or physician? or practitioner?)).mp. (33986)
20 Group Practice/ (6975)
21 or/1-20 (164497)
22 Teaching Materials/ [Educational Materials] (5552)
23 Audiovisual Aids/ (5908)
24 Exhibits as Topic/ (1406)
25 Posters as Topic/ (50)
26 exp Manuals as Topic/ (3351)
27 Textbooks as Topic/ (1699)
28 Medical Illustration/ (3775)
29 Periodicals as Topic/ (31101)
30 Books/ (2109)
31 print$.tw. (16836)
32 paper$.tw. (455505)
33 book?.tw. (15294)
34 monograph?.mp. (2679)
35 pamphlet?.mp. (3716)
36 journal$.tw. (64267)
37 guideline$.mp. (222632)
38 publication$.mp. (58904)
39 (postcard$ or post-card$).mp. (2511)
40 serial$.tw. (93922)
41 textbook$.mp. (6791)

42 manual$.tw. (57787)
43 (hard adj cop$).tw. (839)
44 poster?.tw. (4983)
45 ((static or enduring or educat$ or teach$ or learn$ or instruction$ or train$) adj2 material?).tw. (5287)
46 ((static or enduring or educat$ or teach$ or learn$ or instruction$ or train$) adj2 document?).tw. (246)
47 bulletin?.tw. (2152)
48 message$.tw. (29170)
49 or/22-48 (1007270)
50 (online or on-line).tw. [Delivery Method] (41813)
51 ((web adj page$) or webpage$).tw. (1164)
52 (web-site$ or website$).tw. (12291)
53 cyber$.tw. (2709)
54 exp Online Systems/ (11257)
55 exp Computer-Assisted Instruction/ (8173)
56 exp Internet/ (39324)
57 exp Computers/ (66499)
58 Electronic Mail/ (1462)
59 Telefacsimile/ (209)
60 (handheld adj2 computer$).tw. (343)
61 Internet$.tw. (21894)
62 "personal digital assistant$".tw. (773)
63 (pocket adj PC$).tw. (44)
64 virtual.tw. (22199)
65 (web adj based$).tw. (9756)
66 webbased$.tw. (11)
67 "world wide web$".tw. (2583)
68 www.tw. (1239)
69 (e-bulletin adj board$).tw. (0)
70 "electronic bulletin board$".tw. (52)
71 (electronic adj mail$).tw. (606)
72 email$.tw. (1619)
73 e-mail$.tw. (3768)
74 (list adj serv$).tw. (90)
75 listserv$.tw. (229)
76 (compact adj disc).tw. (205)
77 (compact adj disk).tw. (122)
78 CD-ROM.tw. (1035)
79 DVD.tw. (658)
80 (flash adj drive?).tw. (16)
81 (USB adj key?).tw. (2)
82 (USB adj drive?).tw. (4)
83 workshop?.tw. (22009)
84 ((educat$ or train$ or learn$ or teach$) adj program?).tw. (39153)
85 mail$.tw. (29424)
86 ((educat$ or teach$ or learn$ or instruction$ or train$) and course?).tw. (35705)
87 (fax$ or facsimile?).tw. (1319)

88 disseminat$.tw. (70358)
89 transmit$.tw. (100229)
90 campaign$.tw. (22830)
91 sent.tw. (31413)
92 Postal Service/ (1687)
93 or/50-92 (515888)
94 21 and 49 and 93 (5014)
95 Animals/ not (Animals/ and Humans/) (3557940)
96 94 not 95 (5013)
