# Supplementary material for: What is the effectiveness of printed educational materials on primary care physician knowledge, behaviour, and patient outcomes: a systematic review and meta-analyses
Source: Implement Sci. 2015 Dec 1;10:164. doi: 10.1186/s13012-015-0347-5 (PMC4666153; doi:10.1186/s13012-015-0347-5)
Supplement: Additional file 2: — TIDieR Checklist. Details on reporting of interventions. (PDF 204 kb) [file 13012_2015_347_MOESM2_ESM.pdf]

Study	1. Name	2. Why	3.a) Materials Described	3.b) Materials Available	4. Procedures	5. Provider	6. Mode of Delivery	7. Location	8. When and How Much	9. Tailoring	10. Modifi-cations	11. Planned Adherence	12. Actual Adherence
Kottke, T.E. (1989)	✓	✓	✓	×	×	×	×	✓	✓	N/A	N/A	×	×
Butzlaff, M. (2003)	✓	✓	✓	×	✓	✓	✓	✓	✓	N/A	N/A	✓	✓
Simon, A.E. (2010)	✓	✓	✓	✓	✓	×	✓	✓	✓	N/A	N/A	×	×
Nicholas, J. (2009)	✓	✓	✓	×	✓	✓	✓	✓	✓	N/A	N/A	×	×
Dubey, V. (2006)	✓	✓	✓	✓	✓	✓	✓	✓	✓	N/A	N/A	×	✓
Evans, C.E. (1986)	✓	✓	✓	×	✓	×	×	✓	✓	N/A	N/A	×	×
Hunskaar, S. (1996)	✓	✓	✓	×	✓	×	×	✓	✓	N/A	N/A	×	×
Liaw S.T. (2008)	✓	✓	✓	×	×	×	×	✓	✓	N/A	N/A	×	×
McEwan A. (2002)	✓	✓	×	✓	✓	✓	✓	✓	×	N/A	N/A	×	×
Mukohara, K. (2005)	✓	✓	✓	×	✓	×	✓	×	×	N/A	N/A	✓	✓
Perria C. (2007)	✓	✓	✓	×	×	×	×	✓	×	N/A	N/A	×	×
Tziraki C. (2000)	✓	✓	✓	✓	✓	×	✓	✓	×	N/A	N/A	×	×
Watson M. (2001)	✓	✓	✓	✓	×	×	×	✓	✓	N/A	N/A	×	×
Guadagnoli, E. (2004)	✓	×	✓	×	✓	✓	✓	✓	✓	✓	N/A	×	×
Dickinson, W.P. (2003)	✓	✓	✓	×	✓	×	×	✓	×	N/A	N/A	×	×
Downs, M. (2006)	✓	×	✓	✓	×	×	✓	✓	×	N/A	N/A	×	×
Matowe, L. (2002)	✓	✓	✓	×	✓	✓	✓	✓	✓	N/A	N/A	×	×
Rabin, D. (1994)	✓	✓	✓	×	✓	✓	✓	✓	×	N/A	N/A	×	✓
Rahme, E. (2005)	✓	✓	✓	✓	✓	×	✓	✓	✓	N/A	N/A	×	×
Wright, N.M.J. (2004)	✓	✓	✓	×	✓	✓	✓	✓	✓	N/A	N/A	×	×
Szonyi, G. (1994)	✓	×	✓	✓	✓	×	×	×	×	N/A	N/A	✓	✓
Avorn, J. (1983)	✓	✓	✓	×	✓	×	✓	✓	✓	N/A	N/A	×	×
Bearcroft, P.W.P. (1994)	✓	✓	✓	×	✓	×	×	✓	✓	N/A	N/A	×	×
Dormuth, C.R. (2004)	✓	×	✓	×	✓	×	✓	✓	✓	N/A	N/A	×	×
Oakeshott, P. (1994)	✓	✓	✓	×	✓	×	×	×	✓	N/A	N/A	×	×
Worrall, G. (1999)	✓	✓	×	×	✓	×	✓	×	×	N/A	N/A	×	×
Watson E. (2001)	✓	✓	✓	×	✓	×	×	✓	✓	N/A	N/A	×	×
Hazard, R.G. (1997)	✓	×	✓	×	✓	×	×	✓	✓	N/A	N/A	×	✓
Denig, P. (1990)	✓	✓	✓	×	✓	✓	×	✓	×	N/A	N/A	×	×
Kunz, R. (2007)	✓	✓	✓	✓	✓	✓	✓	✓	✓	✓	N/A	✓	✓
Bjornson, D.C. (1990)	✓	✓	✓	×	✓	✓	×	✓	✓	✓	N/A	✓	✓
Tsuji, S.R. (2007)	✓	×	✓	×	×	×	×	×	×	N/A	N/A	×	×
