# Supplementary material for: What is the effectiveness of printed educational materials on primary care physician knowledge, behaviour, and patient outcomes: a systematic review and meta-analyses
Source: Implement Sci. 2015 Dec 1;10:164. doi: 10.1186/s13012-015-0347-5 (PMC4666153; doi:10.1186/s13012-015-0347-5)
Supplement: Additional file 3: — Study Outcomes. All study outcomes for included studies. (PDF 543 kb) [file 13012_2015_347_MOESM3_ESM.pdf]

Additional file 3: Study-level Outcomes

Clinical Patient Outcomes				
**Author (Year)**	**Outcomes Examined**	**Results**		
		**Control**	**Experiment (PEM)**	**Effect Size and Significance**
Dickinson, W.P. (2003) [21]	1) 12 month intervention effect on physical functioning (SF-36) for multisomatoform disorder, change score (95% CI)	N/A	N/A	5.5 (2.5 to 8.4), effect size: 0.48
	2) 12 month intervention effect on physical functioning (SF-36) for somatization disorder, change score (95% CI)	N/A	N/A	7.1 (3.5 to 10.7), effect size 0.61
	3) 12 month intervention effect on physical functioning (SF-36) for abridged somatization disorder, change score (95% CI)	N/A	N/A	4.2 (1.5 to 6.9), effect size 0.37
	4) 12 month intervention effect for mental health functioning (SF-36) for all 3 somatoform disorders	N/A	N/A	NS
Evans, C.E. (1986) [40]	1) Systolic pressure, mm Hg, Mean (SD)	142.2 (21.0)	140.7 (11.3)	NS
	2) Diastolic pressure, mm Hg, Mean (SD)	88.3 (11.2)	87.6 (5.5)	NS
	3) Patients with a mean diastolic blood pressure of <90 mm Hg, n(%) ** Outcome in M-A	45 (55.6%)	63 (61.8%)	NS
	4) Patients with a minimum diastolic blood pressure of <90 Hg, n(%)	54 (66.7%)	68 (66.7%)	NS
	5) Control of blood pressure by Hypertension Detection and Follow-up Program criteria, n(%)	44 (54.3%)	60 (58.8%)	NS
Hazard, R.G. (1997) [18]	1) No of patients out of work due to back pain, N (%) **Outcome in M-A	6 (24.0%)	8 (28.6%)	NS
Kottke, T.E. (1989) [25]	1) Patient agrees to quit smoking, Average of the proportion of patients for each physician in group (SD)	51.4 (24.9)	61.0 (29.0)	NS
	2) Patient attempts to quit smoking, average % (SD)	44.4 (12.6)	44.0 (9.6)	NS
	3) Patient's duration of cessation, average days (SD)	74.2 (35.8)	66.7 (63.1)	NS
	4) Patient not smoking at time of interview, average % (SD)	14.3 (6.5)	12.0 (7.4)	NS

	5) Cessation verified by cotinine analysis, % **Outcome in M-A	5.0	5.4	NS
Liaw, S.T. (2008) [46]	1) Ownership of a written asthma plan, n (%)	35 (35.0%)	40 (38.1%)	NS
	2. A) Asthma Severity: Moderate/severe	60 (61.2%)	63 (57.3%)	NS
	B) Asthma Severity: Mild	38 (38.8%)	47 (42.7%)	NS
	3) Controlled Asthma, n (%)	57 (58.2%)	73 (66.4%)	NS
	4) Unscheduled visit to GP for asthma attack, n (%)	34 (35.8%)	28 (26.2%)	NS
	5) Visit to ED for asthma attack, n (%)	8 (8.0%)	3 (2.8%)	NS
	6. A) Active quality of life caregiver report, mean (SD)	10.9 (1.8)	11.2 (1.5)	NS
	B) Emotional quality of life caregiver report, mean (SD)	21.5 (3.5)	22.3 (2.9)	NS
	7) Quality of life, self-report of 11-14 year olds, total score, mean (SD)	140.1 (37.6)	148.3 (27.2)	NS
	8. A) Quality of life, self-report of 7-10 year olds: active, mean (SD)	29.0 (3.9)	30.4 (3.1)	NS
	B) Passive, mean (SD)	14.4 (2.1)	17.9 (1.6)	NS
	C) Distress, mean (SD)	30.6 (3.4)	29.9 (4.7)	NS
	D) Severity, mean (SD)	15.7 (4.9)	12.3 (4.2)	NS
	9) Caregiver knowledge, Mean % correct (SD)	66.65 (14.59)	65.29 (12.33)	NS
	10) Adolescent knowledge, Mean % correct (SD)	63.42 (11.73)	60.63 (14.50)	NS
Shah, B. (2014) [42]	1. A) Administrative Data Study Outcomes: Death or non-fatal myocardial infarction (Primary outcome), n/N(%) **Outcome in M-A	11,536/466,076 (2.5%)	11,736/467,713 (2.5%)	OR (95% CI): 1.00 (0.96–1.03), p=0.77
	B) All-cause death (Secondary outcomes – clinical events), n/N(%)	8,704/466,076 (1.9%)	8,704/467,713 (1.9%)	OR (95% CI): 0.98 (0.94–1.01), p=0.21
	C) Myocardial infarction (Secondary outcomes – clinical events), n/N(%)	3,767/466,076 (0.8%)	3,944/467,713 (0.8%)	OR (95% CI): 1.03 (0.97–1.08), p=0.34
	D) Myocardial infarction or unstable angina (Secondary outcomes – clinical events), n/N(%)	4,756/466,076 (1.0%)	5,002/467,713 (1.1%)	OR (95% CI): 1.04 (0.99–1.09), p=0.15
	E) Stroke (Secondary outcomes – clinical events), n/N(%)	1,884/466,076 (0.4%)	1,863/467,713 (0.4%)	OR (95% CI): 0.98 (0.91–1.04), p=0.45
	F) Stroke or transient ischemic attack (Secondary outcomes – clinical events), n/N(%)	2,273/466,076 (0.5%)	2,254/467,713 (0.5%)	OR (95% CI): 0.98 (0.92–1.04), p=0.46

	G) Death, non-fatal myocardial infarction, non-fatal stroke (Secondary outcomes – clinical events), n/N(%)	12,773/466,076 (2.7%)	12,981/467,713 (2.8%)	OR (95% CI): 1.00 (0.97–1.03), p=0.77
	H) Death, non-fatal myocardial infarction, non-fatal stroke, unstable angina, or transient ischemic attack (Secondary outcomes – clinical events), n/N(%)	14,051/466,076 (3.0%)	14,330/467,713 (3.1%)	OR (95% CI): 1.00 (0.96–1.04), p=0.96
	2. A) Clinical Study Data Outcomes: HbA1c<=7.0% (Secondary outcomes – cardiovascular risk reduction), n/N(%)	469/797 (58.8%)	465/795 (58.5%)	OR (95% CI): 0.93 (0.71–1.21), p=0.58
	B) Blood pressure <=130/80 (Secondary outcomes – cardiovascular risk reduction), n/N(%)	506/797 (63.5%)	420/795 (52.8%)	OR (95% CI): 0.72 (0.53–0.98), p=0.04
	C) LDL-cholesterol <=2.0 mmol/l (Secondary outcomes – cardiovascular risk reduction), n/N(%)	492/797 (61.7%)	471/795 (59.2%)	OR (95% CI): 0.90 (0.68–1.18), p=0.43
	D) Total- to HDL-cholesterol ratio <=4.0 (Secondary outcomes – cardiovascular risk reduction), n/N(%)	612/797 (76.8%)	590/795 (74.2%)	OR (95% CI): 0.85 (0.63–1.14), p=0.27
Tsuji, S.R. (2007) [53]	1) Clinical patient remission from depression (HAM-D <8), n/N (%) **Outcome in M-A	65/114 (57%)	84/120 (70%)	p=0.004
Worrall, G. (1999) [37]	1) Patient CES-D score at 6 months, Mean (SD)	19.4 (13.6) (Workshop)	22.2 (11.7)	NS
	2) Patient CES-D gain score at 6 months, Mean (SD)	19.3 (14.6) (Workshop)	15.5 (14.8)	Significant
	3) Physician rating of patient depression at 6 months, Mean (SD)	1.8 (0.7) (Workshop)	2.0 (0.7)	NS
	4) Physician rating of patient depression gain score at 6 months, Mean (SD)	1.1 (0.1) (Workshop)	0.7 (0.1)	Significant
	5) Patients who took antidepressant for full 6 months, No. (%)	42 (46.2) (Workshop)	21 (37.5)	NS
	6) Patients taking medication at 6 months follow-up, No. (%)	51 (56.0) (Workshop)	22 (39.3)	Significant

Physician Behaviour Outcomes		
**Author (Year)**	**Outcomes Examined**	**Results**

		Control	Experiment (PEM)	Effect Size and Significance
Avorn, J. (1983) [19]	1. A) Drugs prescribed: Cephalexin, units	1240	1434	Mean difference: -100; NS
	B) Proxyphene, units	2626	2683	Mean difference: -64; NS
	C) Papaverine, units	1055	953	Mean difference: -104; NS
	D) All 3 drugs, units	4921	5071	Mean difference: -251; NS
Bearcroft, P.W.P. (1994) [30]	1) Requests for chest radiography contrary to guidelines, n(%)	87 (8.2%)	78 (5.7%)	p=0.016
	2) Requests for chest radiography with an inadequate history, n(%)	164 (15.5%)	148 (10.9%)	p=0.0008
	3) Requests for chest radiography that indicated clinical diagnosis, n(%)	454 (42.9%)	668 (49.1%)	p=0.0025
	4) Requests for chest radiography that indicated smoking history	258 (24.4%)	382 (28.0%)	p=0.043
Bishop, P. (2006) [44]	1. A) Patient assessment: History items recorded this episode, %(N)	89% (149)	87% (162)	NS
	B) History items recorded past episode, %(N)	24% (149)	30% (162)	NS
	C) Physical examination findings recorded for regional back exam, %(N)	91% (149)	93% (162)	NS
	D) Physical examination findings recorded for neurological exam, %(N)	63% (149)	63% (162)	NS
	E) Physical examination findings recorded for red flags, %(N)	5% (149)	4% (162)	NS
	2. A) Guideline concordant recommended treatment 0-4 weeks post onset: Education & reassurance, %(N)	7% (149)	10% (162)	NS
	B) Exercise, %(N)	43% (149)	38% (162)	NS
	C) Appropriate medication, %(N)	77% (149)	85% (162)	NS
	D) Spinal manipulation, %(N)	6% (149)	2.5% (162)	NS

	3) Guideline discordant recommended treatment 0-4 weeks post onset: Bedrest >3 days, %(N)	17% (149)	10% (162)	p=0.05
	4. A) Guideline concordant recommended treatment 5-12 weeks post onset for supervised exercise program, %(N)	14% (149)	19% (154)	NS
	B) Return to work, %(N) **Outcome in M-A	17% (149)	24% (154)	NS
	C) Referral to interdisciplinary program, %(N)	2% (149)	4% (154)	Not clear
	5) Guideline discordant recommended treatment 5-12 weeks post onset for physiotherapy >4 weeks, %(N)	43% (149)	41% (154)	0.04
Bjornson, D.C. (1990) [17]	1) Physicians switching patients to both hydralazine and isosorbide, n(%)	N/A	N/A	5 (0.9%) (Control + PEM); p=0.15
	2) Physicians switching patients to at least one of the drugs or discontinued prazosin, n(%)	N/A	N/A	23 (4%) (Control + PEM); p=0.07
Denig, P. (1990) [51]	1. A) Changes in Actual Prescribing of Antispasmodics: Undesirable antispasmodics, Mean daily doses per 1000 prescription (SD)	29.0 (28.3)	25.6 (33.6)	p=0.38
	B) All antispasmodics, Mean daily doses per 1000 prescription (SD)	130.4 (101.2)	115.7 (97.5)	p=0.28
	2. A) Changes in stated prescribing for renal colic: Drugs advised against, Mean daily doses per 1000 prescription (SD)	29.1 (39.1)	17.5 (31.8)	p=0.04
	B) Drugs recommended, Mean daily doses per 1000 prescription (SD)	46.4 (39.2)	51.0 (43.5)	p=0.09
Dormuth, C.R. (2004) [38]	1. A) Number of newly treated patients for analysis drugs where an increase is expected: Cimetidine, N	25	45	RR: 1.53
	B) Metronidazole/(amoxicillin or tetracycline), N	10	9	RR: 2.57
	C) ASA/Ibuprofen/naproxen, N	121	131	RR: 1.26
	D) Isosorbide dinitrate, N	4	7	RR: 1.75
	E) Thiazide diuretics, N	50	69	RR: 1.51

	F) Inhaled corticosteroids, N	4	11	RR: 2.38
	2. A) Number of newly treated patients for analysis drugs where a decrease is expected: Calcium-channel blockers, N	47	38	1/RR: 1.56
	B) Long-acting benzodiazepines, N	191	161	1/RR: 1.22
	C) Hormones, N	87	106	1/RR: 1.04
	D) Calcium-channel blockers, N	65	57	1/RR: 1.34
	E) Clonazepam/alprazolam/diazepam, N	40	47	1/RR: 1.19
	F) Finasteride, N	6	13	1/RR: 0.55
	3) Combined effect of first two outcomes, N **Outcome in M-A	620	748	RR: 1.29
Downs, M. (2006) [33]	1) Diagnosis of dementia concordance scores, Mean (SD)	3.3 (2.0)	3.6 (1.4)	NS
	2) Management of dementia concordance scores, Mean (SD), n **Outcome in M-A	1.3 (1.3), 73	1.5 (1.4), 102	p=0.3
Dubey, V. (2006) [41]	1. A) Rates of preventive manoeuvres: Brushing/flossing, n(%)	298 (6.7)	310 (47.9)	Adjusted RR (95%CI) 9.19 (4.32-19.57)
	B) Blood pressure, n(%)	298 (93.0)	310 (96.6)	1.05 (1.00-1.10)
	C) History of alcohol, n(%)	298 (74.8)	310 (90.9)	1.33 (1.17-1.51)
	D) History of smoking, n(%)	298 (79.2)	310 (92.4)	1.28 (1.16-1.42)
	E) Smoking cessation, n(%)	71 (25.4)	45 (70.0)	3.93 (2.16-7.15)
	F) Tetanus immunization, n(%)	297 (9.4)	308 (40.9)	3.00 (1.72-5.22)
	G) Folic acid counselling, n(%)	125 (3.2)	119 (34.7)	7.47 (2.69-20.75)

	H) Rubella immunity, n(%)	125 (9.6)	119 (34.7)	3.14 (0.78-12.62)
	I) Breast exam, n(%)	148 (77.7)	173 (97.4)	1.06 (0.97-1.16)
	J) Mammography, n(%)	16 (50.0)	50 (76.6)	1.41 (0.76-2.61)
	K) Pap smear, n(%)	145 (88.3)	164 (84.7)	0.92 (0.83-1.01)
	L) Fecal occult blood, n(%)	67 (7.5)	102 (50.6)	6.69 (1.85-24.17)
	M) Hearing assessment, n(%)	33 (12.1)	44 (41.2)	5.13 (0.70-37.32)
	2) Percentage of up to date preventive health services delivered per patient, Mean (%) (95% CI) **Outcome in M-A	48.9 (47.0-50.8)	71.7 (65.1-78.3)	Relative increase of 46.6%, p<0.0001
Evans, C.E. (1986) [40]	1) Patients on blood pressure medication, n(%) **Outcome in M-A	64 (79%)	77 (76%)	NS
	2) Number of medications, Mean (SD)	1.1 (0.74)	1.2 (0.85	NS
	3) Number of tablets per day prescribed, Mean (SD)	1.3 (1.0)	1.1 (0.99)	NS
Feng, B. (2013) [27]	1. A) Behaviours in shared decision making regarding prostate cancer or prostate cancer screening: Overall shared decision-making PCS score, Mean (N)	13.5 (61) (Web course)	10.7 (57)	P<0.05
	B) Provision of information scale, Mean (N)	9.5 (61) (Web course)	8.2 (57)	NS
	C) Elicitation of patient's perspectives scale, Mean (N)	2.5 (61) (Web course)	1.8 (57)	NS
	D) Guiding decision-making scale, Mean (N)	1.5 (61) (Web course)	0.7 (57)	P<0.05
	2. A) Summary of physician final clinical recommendations after prompting by unannounced standardized patient: Recommended in favour of PCS, % (N)	49	68 (57)	NS
	B) Recommended against PCS, % (N)	21 (61) (Web course)	11 (57)	NS

	C) Made no recommendation, % (N)	30 (61) (Web course)	21 (57)	NS
	D) Physician stated that he/she would order a PSA blood test, % (N)	31 (61) (Web course)	60 (57)	P<0.01
French, S. (2013) [47]	1. A) Effect of the intervention on imaging referral: X-ray referral, N	643 (Workshop)	768	Incident rate ratios: 0.83, 95% CI: (0.61, 1.12), p-value: 0.211
	B) CT-scan referral, N	474 (Workshop)	496	0.92, (0.66, 1.27), 0.598
	C) X-ray or CT-scan referral, N **Outcome in M-A	1117 (Workshop)	1264	0.87, (0.68, 1.10), 0.244
Guadagnoli, E. (2004) [22]	1) Provide smoking cessation advice for acute myocardial infarction patients, %(N)	76.1% (67)	67.4% (46)	p=0.31
	2) Advise to restrict salt intake for heart failure patients, %(N)	69.5% (164)	65.4% (159)	p=0.43
	3. A) Diagnosis procedures for patients with acute myocardial infarction: perform cholesterol testing, % (N)	93.1% (277)	92.2% (232)	p=0.70
	B) Determine left ventricular ejection fraction, % (N)	91.7 % (277)	91.8% (232)	p=0.96
	C) Assess symptoms of depression, % (N)	24.5% (277)	24.1% (232)	p=0.91
	4. A) Diagnosis procedures for patients with heart failure: determine left ventricular ejection fraction, % (N)	72.6% (164)	82.4% (159)	p=0.03
	B) Measure serum potassium level, % (N)	88.9% (117)	94.6% (110)	p=0.13
	C) Measure serum creatinine level, % (N)	86.4% (125)	88.0% (117)	p=0.70
	D) Assess patient weight, % (N)	91.5% (164)	94.9% (159)	p=0.21
	E) Assess for peripheral edema, % (N)	94.5% (164)	91.9% (159)	p=0.85
	5) Acute myocardial infarction patients who received care in accordance with treatment recommendations composite score, Mean (no. of patients)	0.77 (277)	0.77 (232)	p=0.81

	6) Heart failure patients who received care in accordance with treatment recommendations composite score, Mean (no. of patients) **Outcome in M-A	0.81 (164)	0.83 (159)	p=0.19
	7. A) Prescribing for patients with acute myocardial infarction: ACE inhibitor, %(N) **Outcome in M-A	66.7% (183)	66.3% (160)	p=0.94
	B) Beta-blocker, %(N)	92.7% (164)	95.1% (141)	p=0.40
	C) Daily aspirin, %(N)	98.5% (258)	99.6% (223)	p=0.23
	8. A) Prescribing for patients with acute myocardial infarction: ACE inhibitor, %(N)	84.6% (39)	90.2% (51)	p=0.42
	B) Target ACE inhibitor, %(N)	46.9% (32)	48.9% (45)	p=0.86
	C) Beta-blocker, %(N)	75.0% (36)	77.1% (48)	p=0.82
Guthrie, B. (2013) [36]	1. A) Change in trend after 2004 risk communication: Oral antipsychotic prescribed, % (95% CI)	N/A	0.54 (20.63 to 20.45)	p<0.001
	B) Oral antipsychotic initiated, % (95% CI)	N/A	0.03 (20.11 to 0.06)	NS
	C) Oral antipsychotic discontinued, % (95% CI)	N/A	0.01 (20.12 to 0.10)	NS
	D) Hypnotic prescribed, % (95% CI)	N/A	0.08 (-0.15 to 0.002)	NS
	E) Anxiolytic prescribed, % (95% CI)	N/A	0.02 (0.09 to 0.05)	NS
	F) Antidepressant prescribed, % (95% CI)	N/A	0.18 (20.37 to 0.02)	NS

	2. A) Change in trend after 2009 risk communication: Oral antipsychotic prescribed, % (95% CI)	N/A	20.51 (20.64 to 20.37)	p<0.001
	B) Oral antipsychotic initiated, % (95% CI)	N/A	20.17 (20.28 to 20.06)	p<0.05
	C) Oral antipsychotic discontinued, % (95% CI)	N/A	0.08 (20.06 to 0.23)	NS
	D) Hypnotic prescribed, % (95% CI)	N/A	20.25 (20.37 to 20.13)	p<0.001
	E) Anxiolytic prescribed, % (95% CI)	N/A	20.37 (20.47 to 20.26)	p<0.001
	F) Antidepressant prescribed, % (95% CI)	N/A	20.69 (20.99 to 20.38)	p<0.001
Kottke, T.E. (1989) [25]	1) Patient reports receiving supportive materials, average proportion of patients for each physician in group (SD)	10.6 (7.7)	36.4 (15.7)	p<0.0001
	2) Patient reports being asked to quit, average proportion of patients for each physician in group (SD)**Outcome in M-A	39.7 (14.2)	54.9 (20.0)	p<0.025
	3) Patient reports being asked for a quit date, average (SD)	5.4 (17.3)	9.6 (19.5)	p<0.005
	4) Patient reports being given follow-up appointment, average (SD)	3.8 (5.5)	6.9 (10.1)	NS
Kunz, R. (2007) [48]	1) Discontinuation of discharge medication, % **Outcome in M-A	29.40%	18.50%	p=0.039
Liaw, S.T. (2008) [46]	1) Use of asthma action plan, n (%)**Outcome in M-A	6 (66.7)	13 (86.7)	p=0.43

Matowe, L. (2002) [32]	1. A) Change in general practitioners' radiography referral trend after 13 months: Total examinations, change in mean before guidelines (95% CI)	N/A	N/A	-1.8 (-11.9, 8.2)
	B) Abdominal ultrasound, change in mean before guidelines (95% CI)	N/A	N/A	<0.1 (-1.1, 1.1)
	C) Ankle X-rays, change in mean before guidelines (95% CI)	N/A	N/A	-0.2 (-0.6, 0.2)
	D) Barium meals, change in mean before guidelines (95% CI)	N/A	N/A	-0.1 (-1.2, 1.1)
	E) Chest X-rays, change in mean before guidelines (95% CI)	N/A	N/A	-0.6 (-4.3, 3.2)
	F) Cervical spine X-ray, change in mean before guidelines (95% CI)	N/A	N/A	-0.6 (-4.3, 3.2)
	G) Foot and toe X-ray, change in mean before guidelines (95% CI)	N/A	N/A	-0.3 (-0.8, 0.1)
	H) Hand and finger X-ray, change in mean before guidelines (95% CI)	N/A	N/A	0.3 (-0.2, 0.7)
	I) Hip X-Ray, change in mean before guidelines (95% CI)	N/A	N/A	-0.4 (-1.0, 0.1)
	J) Kidney, uterus and bladder, change in mean before guidelines (95% CI)	N/A	N/A	0.2 (-0.2, 0.7)
	K) Knee X-Rays, change in mean before guidelines (95% CI)	N/A	N/A	-0.3 (-1.3, 0.6)
	L) Lumbar spine X-rays, change in mean before guidelines (95% CI)	N/A	N/A	-0.4 (-1.2, 0.6)
	M) Pelvic ultrasound, change in mean before guidelines (95% CI)	N/A	N/A	0.2 (-4.5, 0.9)
	N) Pelvis X-ray, change in mean before guidelines (95% CI)	N/A	N/A	-0.3 (-1.5, 0.9)
	O) Shoulder X-rays, change in mean before guidelines (95% CI)	N/A	N/A	<0.1 (-0.4, 0.4)
	P) Sinus X-rays, change in mean before guidelines (95% CI)	N/A	N/A	<0.1 (-0.3, 0.3)
	Q) Testicular ultrasound, change in mean before guidelines (95% CI)	N/A	N/A	<0.1 (-0.5, 0.5)

	R) Thoracic spine X-rays, change in mean before guidelines (95% CI)	N/A	N/A	0.3 (-0.1, 0.7)
McEwan, A. (2002) [35]	1) Rate of opportunistic advice per week, rate (SD) **Outcome in M-A	2.8 (1.8)	4.9 (4.1)	p=0.0025
	2) Rate of giving counselling, rate (SD)	1.0 (1.4)	2.2 (3.2)	p=0.025
	3) Prescribing of nicotine replacement therapy, %	46%	54%	p=0.12
Nicholas, J. (2009) [24]	1. A) Use of BMI percentiles to screen for childhood obesity for age 2-5, Mean (SE) **Outcome in M-A	2.79 (0.17)	3.13 (0.16)	p=0.03
	B) Age 6-11, Mean (SE)	3.00 (0.18)	3.33 (0.16)	p=0.07
	C) Age 12-20, Mean (SE)	3.12 (0.19)	3.46 (0.16)	p=0.08
Oakeshott, P. (1994) [29]	1. A) Radiology requests per practice which conformed to guidelines: Limbs and joints, Mean %, difference from baseline (no. of practices)	83.6, -3.6 (15)	88.8, 3.1 (10)	NS
	B) Chest, Mean %, difference from baseline (no. of practices)	90.6, -2.9 (13)	92.9, 0.7 (13)	NS
	C) Spine, Mean %, difference from baseline (no. of practices)	33.5, -1.2 (14)	44.8, 5.3 (7)	NS
	D) All requests, Mean %, difference from baseline (no. of practices)	73.2, -1.7 (21)	83.5, 10.2 (22)	p<0.01
Perria, C. (2007) [56]	1) Metabolic control (assessment of glycaemic control based on 3 measurements), % (n/N) **Outcome in M-A	10.3 (230/2232)	10.1 (222/2190)	OR (95% CI) for PEM: 0.93 (0.67-1.30)
	2. A) Test for Macrovascular Complications: Total, % (n/N)	12.4 (277/2232)	11.7 (257/2190)	OR (95% CI) for PEM: 0.93 (0.70-1.24)
	B) ECG, % (n/N)	24.2 (541/2232)	25.0 (547/2190)	N/A
	C) Lipid profile, % (n/N)	30.3 (677/2232)	28.5 (624/2190)	N/A
	3. A) Test for Microvascular Complications: Total, % (n/N)	4.7 (105/2232)	4.9 (108/2190)	OR (95% CI) for PEM: 1.11 (0.73-1.69)
	B) Retinal Screening, % (n/N)	22.7 (507/2232)	23.9 (523/2190)	N/A
	C) Microalbuminuaria, % (n/N)	11.9 (265/2232)	10.5 (229/2190)	N/A
	D) Creatinine serum level, % (n/N)	50.4 (1124/2232)	51.6 (1129/2190)	N/A
Rabin, D. (1994) [20]	1. A) Physician reported frequency of questioning new patients about risk factors for sexually transmitted diseases (STDs) and HIV infection: History	-0.06	0.05	p=0.14

	of STD; Mean (SD) before intervention: 3.96 (1.18), Difference between means before and after intervention			
	B) Intravenous drug use; Mean (SD) before intervention: 3.62 (1.33)	0.19	0.34	p=0.28
	C) Condom Use; Mean (SD) before intervention: 3.44 (1.29)	0.19	0.25	p=0.07
	D) Sexual orientation; Mean (SD) before intervention: 3.29 (1.38)	0.16	0.04	p<0.001
	E) Sexual partner's history of intravenous drug use; Mean (SD) before intervention: 2.94 (1.35)	0.07	0.25	p=0.02
	F) Sexual partner’s STD and HIV status; Mean (SD) before intervention: 2.89 (1.25)	0.08	0.23	p=0.34
	G) Anal or oral sex; Mean (SD) before intervention: 2.71 (1.19)	0.08	0.34	p=.008
	H) Number of sexual partner; Mean (SD) before intervention: 2.65 (1.21)	0.23	0.14	p=0.04
	2. A) Physicians observed to conduct risk questioning for sexually transmitted diseases and HIV infection: History of STD (%)	26	35	p=0.14
	B) Intravenous drug use, (%)	15	26	p=0.32
	C) Condom Use, (%)	20	26	p=0.04
	D) Sexual orientation, (%)**Outcome in M-A	9	7	p=0.10
	E) Number of sexual partners, (%)	38	50	p=0.29
	F) Sexual partner's history of intravenous drug use, (%)	32	47	p=0.26
	G) Sexual partner's STD and HIV status, (%)	44	52	p=0.33
	H) Oral sex practices, (%)	8	17	p=0.001
	I) Anal sex practices, (%)	9	19	p=0.002
	3. A) Physician reported frequency of STD and HIV infection prevention advice for women at risk: Condom for vaginal intercourse; Mean (SD) before intervention: 3.97(1.26), Difference between means before and after intervention	0.07	0.05	p=0.001
	B) Condom for anal intercourse; Mean (SD) before	0.11	0.29	p=0.02

	intervention: 3.74(1.69)			
	C) Limit number of partners; Mean (SD) before intervention: 3.48 (1.52)	0.004	0.17	p=0.13
	D) Monogamous; Mean (SD) before intervention: 3.23(1.54)	0.01	0.11	p=0.03
	E) Condom for oral intercourse; Mean (SD) before intervention: 2.69(1.64)	0	0.17	p=0.005
	4. A) Physician observed to provide prevention advice and information on STDs and HIV infection: Use condoms, %	89	88	p=0.04
	B) Use condom for anal sex, %	8	9	p=0.10
	C) Limit number of partners, %	54	54	p=0.99
	D) Be Monogamous, %	49	47	p=0.73
	E) Use condom for oral sex, %	13	19	p=0.08
	F) Educational materials on condoms, %	3	7	p=0.004
Rahme, E. (2005) [39]	1. A) Adequate prescriptions: All prescriptions, n/N (%)	593/1209 (49)	712/1317 (54)	Ratio of odds ratio (95% CI): 1.1 (0.6, 1.6), Probability OR>1=55
	B) Acetaminophen, n/N (%)	399/399 (100)	462/462 (100)	N/A
	C) NSAIDs, n/N (%)	8/123 (7)	10/103 (10)	N/A
	D) COX-2 inhibitors, n/N (%)	186/687 (27)	240/752 (32)	N/A
Shah, B. (2014) [42]	1. A) Administrative data study: Electrocardiogram (Secondary outcomes – Coronary Artery Disease assessment), n/N(%)	187,391/466,076 (40.2%)	181,404/467,713 (38.8%)	OR (95% CI): 0.96 (0.93–0.99), p=0.02
	B) Cardiac stress test or nuclear imaging (Secondary outcomes – Coronary Artery Disease assessment), n/N(%)	37,918/466,076 (8.1%)	36,373/467,713 (7.8%)	0.96 (0.93–1.00), 0.04
	C) Coronary angiography (Secondary outcomes – Coronary Artery Disease assessment), n/N(%)	7,450/466,076 (1.6%)	7,633/467,713 (1.6%)	1.00 (0.96–1.05), 0.83
	D) Cardiology or internal medicine visit (Secondary outcomes – Coronary Artery Disease assessment), n/N(%)	98,944/466,076 (21.2%)	97,193/467,713 (20.8%)	0.97 (0.94–1.00), 0.07
	E) ACEI/ARB (Secondary outcomes – medication initiation), n/N(%)	6,843/56,657 (11.2%)	6,462/58,478 (11.1%)	0.99 (0.95–1.03), 0.65

	F) <=1 antihypertensive class (Secondary outcomes – medication initiation), n/N(%)	4,403/31,133 (14.1%)	4,451/31,825 (14.0%)	0.99 (0.94–1.04), 0.64
	G) <=2 antihypertensive classes (Secondary outcomes – medication initiation), n/N(%)	7,463/77,152 (9.7%)	7,712/79,129 (9.7%)	1.01 (0.97–1.05), 0.59
	H) <=3 antihypertensive classes (Secondary outcomes – medication initiation), n/N(%)	8,176/140,271 (5.8%)	8,377/143,563 (5.8%)	1.00 (0.96–1.04), 0.94
	I) Statin (Secondary outcomes – medication initiation), n/N(%)	7,967/63,891 (12.5%)	8,091/65,391 (12.4%)	1.00 (0.96–1.04), 0.94
	J) Glucose-lowering drug (Secondary outcomes – medication initiation), n/N(%)	6,123/81,047 (7.6%)	6,261/81,565 (7.7%)	1.02 (0.98–1.07), 0.37
	K) Insulin (Secondary outcomes – medication initiation), n/N(%)	3,945/183,622 (2.1%)	4,085/185,311 (2.2%)	1.02 (0.97–1.08), 0.44
	L) Nitrate (Secondary outcomes – medication initiation), n/N(%)	8,936/185,310 (4.8%)	8,726/187,950 (4.6%)	0.96 (0.92–1.00), 0.03
	2. A) Clinical study data: Prescription for statin (primary outcome), n/N(%) **Outcome in M-A	725/797 (91.0%)	700/795 (88.1%)	0.73 (0.42–1.26), 0.26
	B) Prescription for ACEI/ARB (Secondary outcomes – cardiovascular risk reduction), n/N(%)	689/797 (86.4%)	671/795 (84.4%)	0.77 (0.51–1.15), 0.20
	C) When HbA1c>8.0% (Secondary outcomes – clinical inertia), n/N(%)	25/192 (13.0%)	20/170 (11.8%)	0.98 (0.48–1.98), 0.95
	D) When blood pressure>140/90 (Secondary outcomes – clinical inertia), n/N(%)	27/371 (7.2%)	21/337 (5.6%)	0.67 (0.25–1.82), 0.43
	E) When LDL-cholesterol>3.0 mmol/l (Secondary outcomes – clinical inertia), n/N(%)	52/115 (45.2%)	54/124 (43.5%)	0.94 (0.53–1.67), 0.83
Tsuji, S.R. (2007) [53]	1) Appropriate treatment (prescribing of antidepressants), n/N(%) **Outcome in M-A	100/114 (57.7%)	119/120 (99.2%)	p=0.154
Tziraki, C. (2000)	1) Practice adherence to nutrition advice guidelines, Mean adherence score (95% CI)	52.3 (51-54)	53 (52-55)	NS
	2) Adherence to nutrition screening, Mean adherence score (95% CI)	20.5 (20-21)	21 (19-23)	NS
Ulbricht, S. (2014) [50]	1) Number of GPs who did not refer patients in case of prescription drug abuse, n(%)	214 (53.9)	230 (50.5)	N/A
	2) Number of GPs who did not provide treatment in case of prescription drug abuse, n(%)**Outcome in M-A	56 (14.1)	60 (13.2)	N/A
Watson, M. (2001) [28]	1) Prescribing of recommended 3 NSAIDs as a % of total NSAID prescribing, Mean (SD) **Outcome in M-	81.2 (3.7)	80.3 (7.2)	Adjusted mean difference (95% CI)

	A			for PEM vs. control: 0.4 (-2.8 to 3.7)
	2) Prescribing of top 5 NSAIDs as a % of total NSAID prescribing, Mean (SD)	91.0 (2.5)	90.4 (3.2)	0.7 (-0.8 to 2.3)
	3) Prescribing of ibuprofen (Defined Daily Doses (DDDs) per 1000 specific therapeutic group age-sex-related prescribing unit (STAR-PU)), Mean (SD)	195 (34)	166 (42)	8.5 (-14.7 to 31.8)
	4) Prescribing of ibuprofen, % of total DDDs, Mean (SD)	27.7 (4.2)	26.0 (7.6)	2.1 (-1.2 to 5.3)
	5) Total volume of NSAID prescribing, DDDs per 1000 STAR-PUs, Mean (SD)	709 (100)	657 (151)	-14 (-69 to 40)
	6) Prescribing of azapropazone, DDDs per 1000 STAR-PUs	6.4 (5.4)	2.5 (1.7)	-1.9 (-4.7 to 0.8)
Worrall, G. (1999) [37]	1) Correct diagnoses of depression, n(%)	53 (93.4%) (Workshop)	84 (94.6%)	NS
	2) Patients prescribed antidepressant on first visit, n(%)	83 (91.2%) (Workshop intervention)	50 (89.3%)	NS
	3. A) Number of referrals to: Psychiatrist, n **Outcome in M-A	6 (workshop)	2	NS
	B) Other mental health professional, n **Outcome in M-A	8 (workshop)	0	NS
Wright, N.M.J. (2004) [31]	1. A) Number of items prescribed monthly: Chlorpromazine hydrochloride, Mean (95% CI)	N/A	160 (-10 to 320)	p=0.06
	B) Haloperidol, Mean (95% CI)	N/A	120 (60 to 180)	p<0.001
	C) Olanzapine, Mean (95% CI)	N/A	60 (-40 to 160),	p=0.2
	D) Risperidone, Mean (95% CI)	N/A	50 (-90 to 180),	p=0.5
	E) Thioridazine, Mean (95% CI)	N/A	-810 (-1200 to -420)	p<0.001
Zwarenstein, M. (2014) [58]	1) Percentage of patients obtaining retinal screening within 90 days of mail out, Median success rate % (25th and 75th percentile success rates) **Outcome in M-A	31.0 (25.0, 37.0)	Insert: 30.9 (25.3, 37.8); Outsert, no reminder	0.96

			notepad: 30.8 (25.0, 37.1); Outsert and reminder pad: 30.4 (25.0, 37.5); Insert and outsert, no reminder notepad: 30.3 (25.0, 37.3); Insert and outsert and reminder notepad: 30.4 (25.0, 37.5); Overall: 30.8 (25.0, 37.5)	

Physician Cognition Outcomes				
**Author (Year)**	**Outcomes Examined**	**Results**		
		**Control**	**Experiment (PEM)**	**Effect Size and Significance**
Butzlaff, M. (2003) [49]	1) Number of participants with knowledge increase between the intervention and control	N/A	N/A	p=0.50
Denig, P. (1990) [51]	1. A) Changes in perceived drug utility, on a scale of 1-10: Butylscopolamine suppositories (advised against) for irritable bowel syndrome, Mean (SD)	5.1 (2.6)	4.7 (2.9)	p=0.23
	B) Chlordiazepoxide combination tablets (advised against) for irritable bowel syndrome, Mean (SD)	4.3 (2.3)	3.9 (2.6)	p=0.22
	C) Metamizole combination tablets (advised against) for renal colic, Mean (SD)	6.6 (2.5)	5.5 (2.8)	p=0.03
	D) Diclofenac injections (recommended) for renal colic, Mean (SD)	7.2 (1.8)	7.4 (1.8),	p=0.15

	2. A) Changes in knowledge: Improved knowledge on IBS, # of physicians (%)	9 (20.9%)	11 (26.8%)	p>0.05
	B) Improved knowledge on Renal Colic, # of physicians (%) **Outcome in M-A	9 (37.5%)	20 (81.4%)	p<0.02
French, S. (2013) [47]	1. A) Effect of the intervention on clinical behaviours as measured by response to vignettes: X-ray adherence, n/N	126/152 (Workshop)	109/160	Adjusted odds ratio: 1.76, 95% CI: (1.01, 3.05), p-value: 0.045
	B) Imaging adherence, n/N	119/152 (Workshop)	89/160	2.36 (1.48, 3.79), 0.000
	C) Activity adherence, n/N	121/152 (Workshop)	82/160	4.49, (1.90, 10.60), 0.001
	D) Bed rest adherence, n/N	163/164 (Workshop)	168/171	2.91, (0.30, 27.83), 0.354
Hunskaar, S. (1996) [55]	1. Views on prescribing oestrogen based on case history number 1-9, median (interquartile range)	1) 4(3-4), 2) 3(2-4), 3) 2(1-3), 4) 3(2-4), 5) 3(1-3), 6) 4(4-5), 7) 4(3-5), 8) 5(4-5), 9) 4(3-4)	1) 4(3-4), 2) 3(2-4), 3) 2.5(1-3), 4) 3(2-4), 5) 3(1.5-3), 6) 5(4-5), 7) 4(3-5), 8) 5(4-5), 9) 4(3-5)	NS
Liaw, S.T. (2008) [46]	1) Identification of a child with asthma that may be high risk, % of correct responses out of 10, mean (SD)	82.2 (8.3)	89.3 (9.6)	Significant
	2) Assessment of severity of acute attack, % of correct responses out of 11, mean (SD)	33.8 (13.3)	40.1 (17.3)	p=0.05
	3. A) Confidence in management of: Acute attack, n(%)	5 (55.6) Very confident, 3 (33.3) Confident, 1 (11.1) Not very confident	12 (80.0) Very confident, 3 (20.0) Confident	p=0.09
	B) Moderate acute attack, n(%)	4 (50.0) Very confident, 3 (37.5)	11 (78.6) Very confident, 3	p=0.30

		Confident, 1 (12.5) Not very confident	(21.4) Confident;	
	C) Severe/critical attack, n(%)	3 (33.3) Very confident, 3 (33.3) Confident, 3 (33.3) Not very confident	3 (20.0) Very confident, 7 (46.7) Confident, 5 (33.3) Not very confident	p=0.58
	4. A) Ongoing management of: Infrequent episodic asthma, n(%)	3 (33.3) Very confident, 5 (55.6) Confident, 11 (11.1) Not very confident	9 (60.0) Very confident, 6 (40.0) Confident	p=0.03
	B) Frequent episodic asthma, n(%)	3 (33.3) Very confident, 5 (55.6) Confident, 1 (11.1) Not very confident	CPG: 6 (40.0) Very confident, 9 (60.0) Confident	p=0.49
	C) Persistent asthma, n(%)	3 (33.3) Very confident, 5 (55.6) Confident, 1 (11.1) Not very confident	5 (33.3) Very confident, 8 (53.3) Confident, 2 (13.3) Not very confident	p=0.73
	5) Knowledge about asthma, % correct out of 21, Mean (SD) **Outcome in M-A	70.2 (14.5)	71.1 (11.6) CPG	p=0.06
Mukohara, K. (2005) [26]	1. A) Self-reported skills: Critical appraisal, Mean change (95% CI)	0.07 (-0.02 to 0.17)	0.07 (0.00 to 0.13)	p=0.9
	B) Evidence-based medicine quantitative skills, Mean change (95% CI)	0.1 (-0.05 to 0.3)	0.1 (-0.01 to 0.2)	p=0.9
Secher, N. (2012) [52]	1. A) Correct answers to questions on basic life support and the use of an automated external defibrillator: Diagnosis of a cardiac	70 (27.7%)	58 (26.8%)	p=0.84

	arrest, n(%)			
	B) The first action to take when a person has cardiac arrest in your clinic, n(%)	79 (31.3%)	73 (33.7%)	p=0.62
	C) Recommended compression depth, n(%)	100 (39.5%)	143 (66.2%)	p=<0.001
	D) Recommended compression frequency, n(%)	95 (37.7%)	133 (61.6%)	p=<0.001
	E) Recommended compression ventilation ratio, n(%)	176 (69.8%)	162 (75.0%)	p=0.25
	F) Recommended volume for ventilation, n(%)	231 (91.7%)	190 (88.0%)	p=0.22
	G) When to place a person in the recovery position, n(%) **Outcome in M-A	240 (95.2%)	200 (92.6%)	p=0.25
	H) When to use an automated external defibrillator at a cardiac arrest, N(%)	220 (87.3%)	184 (85.2%)	p=0.67
	I) How to place the automated external defibrillator pads, n(%)	224 (88.9%)	183 (84.7%)	p=0.22
	2. A) Self-evaluation skills: "I feel confident performing basic life support", mean Likert score (SD), %	4.4 (0.6), 94.4%	4.4 (0.6), 95.8%	p=0.81
	B) "I feel I can handle a person with cardiac arrest in my clinic", mean Likert score (SD), %	4.1 (0.7), 87.7%	4.2 (0.7), 88.8%	p=0.38
	C) "I think more BLS training relevant for general physicians are needed", mean Likert score (SD), %	3.5 (1.1,) 55.4%	3.6 (1.0), 59.7%	p=0.50
Simon A.E. (2010) [54]	1. A) Diagnosis test questionnaire with all responses included at 6 months, mean score (n): A) at 6 months **Outcome in M-A	5.63 (149)	6.64 (176)	p<0.001
	B) Diagnosis test questionnaire with all responses included at 12 months, mean score (n)	5.53 (146)	6.06 (174)	p=0.046
Szonyi, G. (1993) [45]	1) Score on incontinence knowledge questionnaire, scored with negative marking, % score (SD) **Outcome in M-A	33 (17)	60 (19)	p<0.001
Watson, E. (2001) [34]	1) GPs making correct referral decisions on at least 5 of 6 family history vignettes, %(n) **Outcome in M-A	38.9 (63)	80.6 (100)	p<0.001
