# Supplementary material for: What is the effectiveness of printed educational materials on primary care physician knowledge, behaviour, and patient outcomes: a systematic review and meta-analyses
Source: Implement Sci. 2015 Dec 1;10:164. doi: 10.1186/s13012-015-0347-5 (PMC4666153; doi:10.1186/s13012-015-0347-5)
Supplement: Additional file 4: — Risk of Bias. Appraisal of risk of bias of included RCT studies using Cochrane EPOC risk of bias assessment tool. (PDF 141 kb) [file 13012_2015_347_MOESM4_ESM.pdf]

Additional file 4: Appraisal of Risk of Bias of Included RCT studies Using Cochrane EPOC Risk of Bias Assessment Tool

	Risk of Bias								
Study	Allocation generation	Allocation concealment	Baseline outcome measurement	Baseline characteristic measurement	Incomplete outcome data	Knowledge of allocated interventions	Protection against contamination	Selective outcome reporting	Other
Avorn, J. (1983)	Unclear	Low	Low	Unclear	Low	Low	Low	Low	High
Bearcroft, P.W.P. (1994)	Low	Low	Unclear	High	Unclear	Low	Low	Low	Low
Bishop, P. (2006)	Low	Unclear	Unclear	Unclear	Low	Unclear	High	Unclear	High
Bjornson, D.C. (1990)	Unclear	Unclear	Unclear	Unclear	High	High	Unclear	Low	Low
Butzlaff, M. (2003)	Low	Low	Low	Unclear	Low	High	Low	Low	Low
Denig, P. (1990)	Unclear	High	High	Unclear	Low	High	Low	Low	Low
Dickinson, W.P. (2003)	Unclear	High	Low	Low	Low	Unclear	High	High	High
Dormuth, C.R. (2004)	Low	Low	Unclear	Low	Low	Low	Low	Low	Low
Downs, M. (2006)	Low	Low	Low	High	Low	Unclear	Low	Low	High
Dubey, V. (2006)	Low	Low	Low	Low	Low	Low	Low	Low	Low
Evans, C.E. (1986)	Unclear	Low	Low	Unclear	Low	Low	Low	High	Low
Feng, B. (2013)	Unclear	Unclear	High	Unclear	Unclear	Low	Unclear	Low	High
French, S. (2013)	Low	Low	Unclear	High	Low	Low	Low	Low	Low
Guadagnoli, E. (2004)	Low	Low	Unclear	Low	Low	Unclear	Unclear	Low	High
Hazard, R.G. (1997)	Low	High	Unclear	High	Low	High	High	High	High
Hunskaar, S. (1996)	Low	Low	High	High	Unclear	High	Unclear	Low	High
Kottke, T.E. (1989)	Unclear	Low	High	High	Unclear	Unclear	Low	Unclear	Low
Kunz, R. (2007)	Low	Low	Unclear	High	High	High	Low	Low	High
Liaw S.T. (2008)	Low	Low	Low	High	Low	High	Low	Low	Low
McEwan A. (2002)	Unclear	Low	Unclear	Low	Low	High	Low	Low	High
Mukohara, K. (2005)	Low	High	Low	Low	Low	High	Unclear	Low	Low
Nicholas, J. (2009)	Unclear	Unclear	Low	Low	Low	High	Unclear	Low	Low
Oakeshott, P. (1994)	Unclear	Low	Unclear	High	Low	Low	Low	Low	Low
Perria C. (2007)	Low	Low	Low	Low	High	Low	Low	High	Low
Rabin, D. (1994)	Low	High	Low	Low	Low	Unclear	High	Unclear	High
Rahme, E. (2005)	Unclear	Unclear	Low	Low	Low	High	Low	Low	High
Secher, N. (2012)	Low	Low	Unclear	Low	Low	High	Low	Low	High
Shah, B. (2014)	Low	Low	Low	Low	Low	Low	Low	Low	Low
Simon, A.E. (2010)	High	Unclear	Low	High	Low	High	High	High	Low
Szonyi, G. (1994)	Low	Unclear	Low	High	High	Unclear	Unclear	Low	High
Tsuji, S.R. (2007)	Unclear	Low	High	Low	Low	Low	Low	Low	Low
Tziraki C. (2000)	Unclear	Low	Unclear	Low	Unclear	High	Low	Low	Low
Ulbricht, S. (2014)	Unclear	Unclear	Unclear	Low	Unclear	High	Unclear	Low	High
Watson E. (2001)	Low	Low	Unclear	High	High	High	Low	Low	Low
Watson M. (2001)	Unclear	Low	High	Unclear	High	Low	Low	Low	Low
Worrall, G. (1999)	Low	Low	Low	Unclear	Low	High	Low	Low	High
Zwarenstein, M. (2014)	Low	Low	Unclear	Low	Low	Low	Low	Low	Low
