# Supplementary material for: What is the effectiveness of printed educational materials on primary care physician knowledge, behaviour, and patient outcomes: a systematic review and meta-analyses
Source: Implement Sci. 2015 Dec 1;10:164. doi: 10.1186/s13012-015-0347-5 (PMC4666153; doi:10.1186/s13012-015-0347-5)
Supplement: Additional file 5: — Meta-Analysis Results. Forest Plots for Meta-Analyses. (PDF 395 kb) [file 13012_2015_347_MOESM5_ESM.pdf]

Meta-Analyses Results

A) Patient Dichotomous Outcomes

Author and Year	Outcome	Intervention Events	Intervention N	Control Events	Control N	Weight	RR [95% CI]
Kottke, 1989	Verified smoking cessation at one year follow-up	32	593	20	400	0 %	1.08 [0.24 , 4.92]
Evans, 1986	Control of Hypertension by Mean DBP < 90 mm Hg	63	102	45	81	0 %	1.11 [0.86 , 1.44]
Hazard, 1997	Return to work after back injury at 3 months	20	28	19	25	0 %	0.94 [0.68 , 1.30]
Tsuji, 2009	Patient remission from depression	84	120	65	114	0 %	1.23 [0.87 , 1.73]
Shah, 2014	Survived with no myocardial infarction	455977	467713	454540	466076	100 %	1.00 [1.00 , 1.00]
Overall, $I^2 = 0\%$						100%	1.00 [1.00 , 1.00]

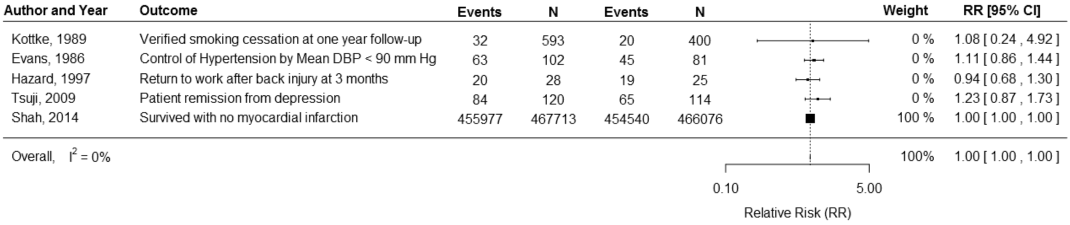

Favours Control *Favours Intervention*

B) Patient Dichotomous Outcomes (with Shah 2014 removed)

Author and Year	Outcome	Intervention Events	Intervention N	Control Events	Control N	Weight	RR [95% CI]
Kottke, 1989	Verified smoking cessation at one year follow-up	32	593	20	400	1.3 %	1.08 [0.24 , 4.92]
Evans, 1986	Control of Hypertension by Mean DBP < 90 mm Hg	63	102	45	81	44.17 %	1.11 [0.86 , 1.44]
Hazard, 1997	Return to work after back injury at 3 months	20	28	19	25	28.94 %	0.94 [0.68 , 1.30]
Tsuji, 2009	Patient remission from depression	84	120	65	114	25.59 %	1.23 [0.87 , 1.73]
Overall, $I^2 = 0\%$						100%	1.09 [0.91 , 1.29]

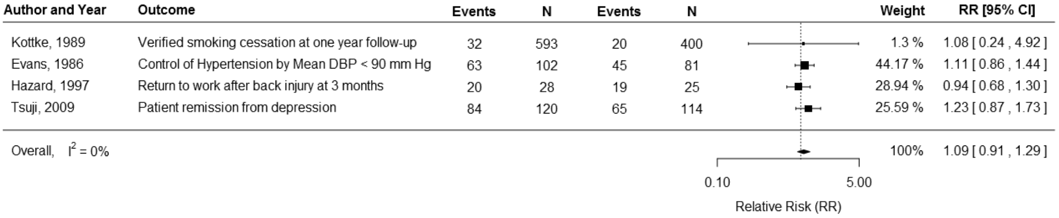

Favours Control *Favours Intervention*

C) Physician Behaviour Continuous Outcomes (N=physicians)

Author and Year	Outcome	Intervention N	Intervention Mean	Intervention SD	Control N	Control Mean	Control SD	Weight	SMD [95% CI]
McEwan, 2002	Provide opportunistic advice to stop smoking	37	4.9	4.1	37	2.8	1.8	24.39 %	0.66 [0.16 , 1.15]
Nicholas, 2009	Using BMI to screen for childhood obesity	84	3.13	1.47	76	2.79	1.48	31.05 %	0.23 [-0.08 , 0.54]
Kottke, 1989	Ask to quit smoking	22	54.9	20	17	39.7	14.2	19.06 %	0.84 [0.18 , 1.50]
Watson, 2001	Prescribing of recommended NSAIDs	36	80.3	7.2	36	81.2	3.7	25.5 %	-0.16 [-0.62 , 0.31]
Overall, $I^2 = 67.88\%$								100%	0.35 [-0.06 , 0.76]

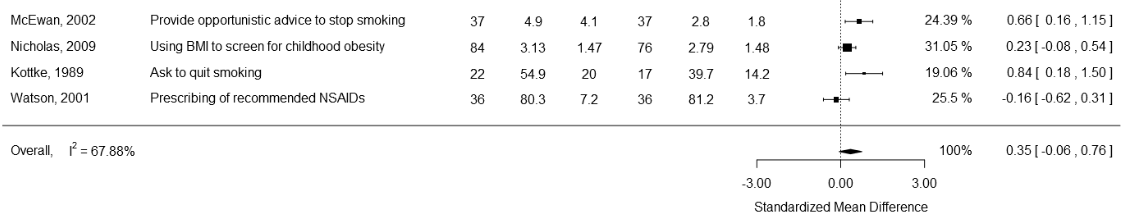

Favours Control *Favours Intervention*

D) Physician Behaviour Continuous Outcomes (N=patients)

Author and Year	Outcome	Intervention N	Intervention Mean	Intervention SD	Control N	Control Mean	Control SD	Weight	SMD [95% CI]
Downs, 2006	Best practice dementia management	102	1.5	1.4	73	1.3	1.3	13.48 %	0.14 [-0.59 , 0.87]
Guadagnoli, 2004	Recommended practice for newly discharged patients with HF	159	0.83	0.13	164	0.81	0.19	49.84 %	0.12 [-0.10 , 0.34]
Dubey, 2006	Percentage of up to date preventive health services delivered*	310	71.7	59.29	298	48.9	16.73	36.67 %	0.52 [0.18 , 0.85]
Overall, $I^2 = 68.74\%$								100%	0.27 [-0.03 , 0.57]

HF=Heart Failure
* Percentage delivered per patient

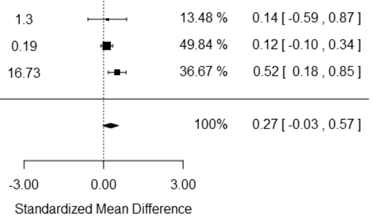

Favours Control *Favours Intervention*

D) Physician Behaviour Dichotomous Outcomes (N=physicians)

Author and Year	Outcome	Intervention Events	Intervention N	Control Events	Control N	Weight	RR [95% CI]
Ulbricht, 2014	Provision of treatment in case of prescription drug dependency	395	455	341	397	98.7 %	1.01 [0.96 , 1.07]
Liaw, 2008	Physician use of asthma action plan	13	15	6	9	1.1 %	1.30 [0.78 , 2.16]
Rabin, 1994	Physicians observed to conduct risk questioning for STDs & HIV*	4	58	6	65	0.19 %	0.75 [0.22 , 2.52]
Overall, $I^2 = 0\%$						100%	1.01 [0.96 , 1.07]

* Questioning was done on sexual orientation

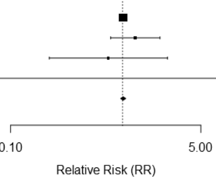

Favours Control *Favours Intervention*

F) Physician Behaviour Dichotomous Outcomes N=patients)

Author and Year	Outcome	Intervention Events	Intervention N	Control Events	Control N	Weight	RR [95% CI]
Bishop, 2006	Recommendation to return to work 5-12 weeks after onset of low back pain	37	154	25	149	0.21 %	1.43 [0.91 , 2.26]
Guadagnoli, 2004	Prescribe ACE inhibitor for newly discharge patients with MI	106	160	122	183	1.92 %	0.99 [0.85 , 1.16]
Evans, 1986	Patients on blood pressure medication	77	102	64	81	1.66 %	0.96 [0.81 , 1.12]
Kunz, 2007	Continuation of discharge medication	140	172	68	96	1.96 %	1.15 [0.99 , 1.33]
Dormuth, 2004	Evidence-based drug prescribing	748	1661773	620	1494941	0 %	1.07 [0.00 , 824.66]
Tsuji, 2009	Prescription of antidepressants	119	120	100	114	9.8 %	1.00 [0.94 , 1.07]
Shah, 2014	Prescription for statin	700	795	725	797	11.67 %	0.97 [0.91 , 1.03]
Zwarenstein, 2014	Retinal screening within 90 days	16809	53768	17683	55961	72.17 %	0.99 [0.97 , 1.01]
Perria, 2007	Assessment of glycaemic control based on 3 measurements	222	2190	230	2232	0.59 %	0.98 [0.75 , 1.29]
Overall, $I^2 = 0\%$						100%	0.99 [0.97 , 1.01]

MI = Myocardial infarction

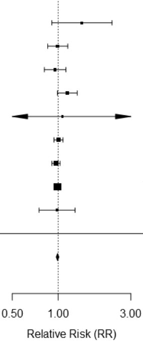

Favours Control *Favours Intervention*

G) Physician Behaviour Dichotomous Outcomes, PEMs vs. Workshop

Author and Year	Outcome	Intervention Events	Intervention N	Control Events	Control N	Weight	RR [95% CI]
French, 2013	X-Ray or CT-Scan referrals	1264	75226	1117	77716	55 %	1.17 [0.38 , 3.62]
Worral, 1999	Referrals to psychiatrist or other mental health professional	2	56	14	91	45 %	0.23 [0.05 , 1.05]
Overall, $I^2 = 64.48\%$						100%	0.57 [0.12 , 2.73]

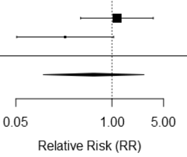

Favours Control *Favours Intervention*

H) Physician Cognition Continuous Outcomes

Author and Year	Outcome	Intervention N	Intervention Mean	Intervention SD	Control N	Control Mean	Control SD	Weight	SMD [95% CI]
Liaw, 2008	Knowledge on asthma, questionnaire score	15	71.1	11.6	9	70.2	14.5	26.87 %	0.07 [-0.86 , 0.99]
Szonyi, 1994	Knowledge on incontinence, questionnaire score	48	60	19	41	33	17	34.99 %	1.48 [1.01 , 1.95]
Simon, 2010	Knowledge on diagnosing somatization at 6 months	176	6.64	3.45	149	5.63	3.17	38.14 %	0.30 [0.08 , 0.52]
Overall, $I^2 = 89.75\%$								100%	0.65 [-0.21 , 1.51]

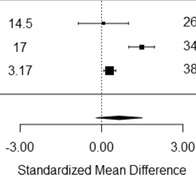

Favours Control *Favours Intervention*

<u>I) Physician Cognition Dichotomous Outcomes</u>

Author and Year	Outcome	Intervention Events	Intervention N	Control Events	Control N	Weight	RR [95% CI]
Secher, 2012	Knowledge of when to place a person into recovery position*	200	216	240	252	38.96 %	0.97 [0.92 , 1.03]
Denig, 1990	Improved knowledge on renal colic	20	28	9	24	26.49 %	1.90 [1.08 , 3.36]
Watson, 2001	GPs making correct referral decision on family history vignettes	100	124	63	162	34.55 %	2.07 [1.54 , 2.80]
Overall, I^2 = 90.27%						100%	1.51 [0.90 , 2.52]

* During basic life support (correct answers)

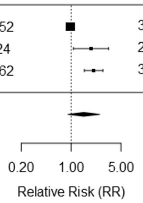

Favours Control *Favours Intervention*
