# Supplementary material for: What is the effectiveness of printed educational materials on primary care physician knowledge, behaviour, and patient outcomes: a systematic review and meta-analyses
Source: Implement Sci. 2015 Dec 1;10:164. doi: 10.1186/s13012-015-0347-5 (PMC4666153; doi:10.1186/s13012-015-0347-5)
Supplement: Additional file 6: — Sub-analyses for Heterogeneity. Results of sub-analyses for heterogeneity. (PDF 140 kb) [file 13012_2015_347_MOESM6_ESM.pdf]

Additional file 6: Sub Analyses for Heterogeneity

Outcome	Number of Studies	**Summary Statistic** RR=Relative Risk SMD=Standard Mean Difference	Confidence Interval	I^2 statistic
Physician Behaviour Outcomes (N=physician): Sub Analysis for Similar Topic	2 [25, 35]	SMD=0.72	0.34-1.10	0%
Physician Behaviour Outcomes (N=physician): Sub Analysis for Similar Type of Intervention	2 [24, 28]	SMD=0.08	-0.29-0.45	45%
Physician Behaviour Outcomes (N=physician): Sub Analysis for Similar Length of Intervention	2 [24, 25]	SMD=0.46	-0.12-1.04	63%
Physician Behaviour Outcomes (N=physician): Sub Analysis for Similar Participants	3 [25, 28, 35]	SMD=0.42	-0.18-1.03	75%
Physician Behaviour Outcomes (N=physician): Sub Analysis for Similar Behaviour Targeted	2 [25, 35]	SMD=0.72	0.34-1.10	0%
Physician Behaviour Outcomes (N=patient): Sub Analysis for Similar Type of Intervention	2 [22, 41]	SMD=0.33	-0.06-0.72	88%
Physician Behaviour Outcomes (N=patient): Sub Analysis for Similar Risk of Bias	2 [33, 41]	SMD=0.35	-0.01-0.72	78%
Physician Behaviour Outcomes (N=patient): Sub Analysis for Similar Targeted Behaviour	2 [22, 33]	SMD=0.13	-0.05-0.31	0%
